# Combining experiments on luminescent centres in hexagonal boron nitride with the polaron model and *ab initio* methods towards the identification of their microscopic origin†

**DOI:** 10.1039/d3nr01511d

**Published:** 2023-08-16

**Authors:** Moritz Fischer, Ali Sajid, Jake Iles-Smith, Alexander Hötger, Denys I. Miakota, Mark K. Svendsen, Christoph Kastl, Stela Canulescu, Sanshui Xiao, Martijn Wubs, Kristian S. Thygesen, Alexander W. Holleitner, Nicolas Stenger

**Affiliations:** a Department of Electrical and Photonics Engineering, Technical University of Denmark 2800 Kgs. Lyngby Denmark niste@dtu.dk; b Centre for Nanostructured Graphene, Technical University of Denmark 2800 Kgs. Lyngby Denmark; c NanoPhoton – Center for Nanophotonics, Technical University of Denmark 2800 Kgs. Lyngby Denmark; d Department of Physics, Technical University of Denmark 2800 Kgs. Lynby Denmark; e Department of Electrical and Electronic Engineering, The University of Manchester Sackville Street Building Manchester M1 3BB UK; f Walter Schottky Institute and Physics Department, Technical University of Munich 85748 Garching Germany; g School of Physics and Astronomy Monash University Victoria 3800 Australia

## Abstract

The two-dimensional material hexagonal boron nitride (hBN) hosts luminescent centres with emission energies of ∼2 eV which exhibit pronounced phonon sidebands. We investigate the microscopic origin of these luminescent centres by combining *ab initio* calculations with non-perturbative open quantum system theory to study the emission and absorption properties of 26 defect transitions. Comparing the calculated line shapes with experiments we narrow down the microscopic origin to three carbon-based defects: C_2_C_B_, C_2_C_N_, and V_N_C_B_. The theoretical method developed enables us to calculate so-called photoluminescence excitation (PLE) maps, which show excellent agreement with our experiments. The latter resolves higher-order phonon transitions, thereby confirming both the vibronic structure of the optical transition and the phonon-assisted excitation mechanism with a phonon energy ∼170 meV. We believe that the presented experiments and polaron-based method accurately describe luminescent centres in hBN and will help to identify their microscopic origin.

## Abstract 2

We narrow down the microscopic origin to 3 out of 26 defect transitions by comparing experiments with *ab initio* methods and the polaron model. We show excellent agreement between theory and experimental photoluminescence excitation spectroscopy.

## Graphical abstract

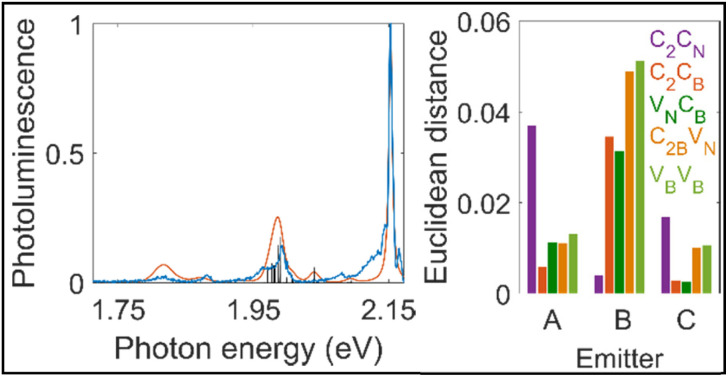

## Introduction

Luminescent centres in hexagonal boron nitride (hBN) have gained an increased scientific interest due to the demonstration of single photon emission with a brightness comparable to semiconductor quantum dots.^1^ These luminescent centres emit at photon energies around 2 eV and persist even at room temperature,^2^ making hBN a promising material to realise future optoelectronic technologies such as quantum telecommunication^3,4^ and quantum sensing.^5^

The microscopic origin of 2 eV luminescent centres in hBN has been experimentally narrowed down to carbon-based defects by bottom-up and post-growth techniques,^6^ however the atomic structure of the underlying defect remains elusive. One subset of 2 eV luminescent centres are group I centres^7^ with pronounced phonon side bands (PSB). The chemical composition of the defect will naturally alter the mechanical vibrations of the crystal which will in-turn modify the structure of the PSB observed in photoluminescence. This motivates the comparison of experimental lineshapes with those obtained *via ab initio* methods.^8–10^

A typical approach to calculating the photoluminescence of defect transitions is to first calculate the electronic structure and phonon modes using *ab initio* methods, before using this information to calculate the photoluminescence spectrum with the generating function approach.^8–14^ Whilst this method can calculate the linear absorption and emission spectra with atomistic precision, it cannot resolve coherent dynamics between electronic states of the defect transition, and is limited to unstructured electromagnetic environments. As the field strives towards coherent control of hBN luminescent centres,^15^ and interfacing emitters with plasmonic^16^ and photonic structures,^17^ new theoretical methods are required to describe the behaviour of defect complexes.

In this joint experiment-theory investigation, we study the photoluminescence and photoluminescence emission (PLE) of 2 eV luminescent centres in hBN. We combine a non-perturbative master equation treatment of electron–phonon interaction with *ab initio* methods to calculate the photoluminescence line shapes of 26 candidate defect transitions. This methodology, based on the polaron formalism,^18^ maintains the atomistic accuracy of the generating function approach, while allowing to simulate PLE maps of the studied defect transition by taking into account external driving fields. By comparing the calculated photoluminescence against measurements of twelve group I centres we exclude all but three candidate defects: the neutral substitutional carbon trimers C_2_C_B_^8,9^ and C_2_C_N_^8,9,19^ as well as V_N_C_B_.^7,10,20^ Here, C_2_C_B_ and C_2_C_N_ are shorthand notations of C_B_C_N_C_B_ and C_N_C_B_C_N_, respectively. By comparing measured and theoretical PLE, which may be calculated directly using the polaron formalism, we are able to resolve higher-order phonon transitions. By studying the emission of zero-phonon line (ZPL) and PSB simultaneously, we confirm that the excitation mechanism in group I emitters is phonon-assisted with a phonon energy of ∼170 meV. The combination of theoretical and experimental methods presented here will help to identify the microscopic origin of luminescent centres in hBN.

## Results and discussion

To generate luminescent centres in hBN we use a process that was developed recently in our group.^7^ Briefly, we use bulk hBN crystals and mechanically exfoliate them with tape onto SiO_2_/Si substrates with a 100 nm oxide thickness. The typical flakes studied in this work showed thicknesses ranging from 15 nm to 30 nm, which corresponds approximately to 50 and 100 layers, respectively. We then irradiate our high-quality multilayer hBN with oxygen atoms and use subsequent annealing to achieve high densities of luminescent centres emitting at photon energies around 2 eV. In our previous work, we carried out room-temperature characterisation of group I and group II centres, as defined in ref. 7. In the work presented here, we focus our attention on low-temperature characterisation of group I centres which show a specific line shape with pronounced PSB at ZPL detunings around 170 meV, as observed also in other works.^21–23^ Furthermore, these group I centres emit at photon energies around 2 eV just like single-photon emitters in hBN.^2^ In this work, we define luminescent centres as experimental line shapes while theoretical line shapes are called defect transitions. Single-photon emitters are luminescent centres with an auto-correlation function fulfilling the criterion *g*^(2)^(0) < 0.5. In the following we will study luminescent centres with a focus on group I line shapes.

**Fig. 1:**
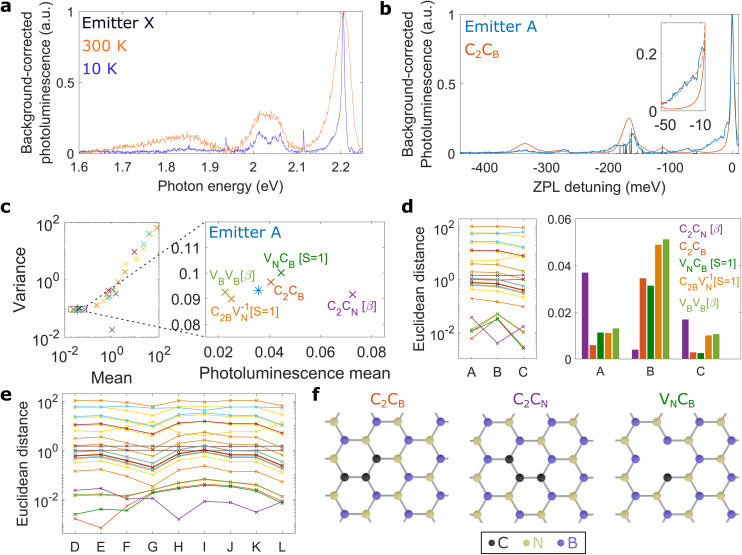
Screening of defect transitions. (a) Room- and low-temperature spectra of emitter X under 2.37 eV excitation. a.u., arbitrary units. (b) Comparison of low-temperature photoluminescence of emitter A (under 2.321 eV excitation) with the theoretical emission line shape of C_2_C_B_ using the PBE functional (without acoustic phonons). The black bars show the partial Huang–Rhys factors (a.u.) obtained with the PBE functional. The inset shows the theoretical line shape of C_2_C_B_ using the HSE06 functional with and without acoustic phonons in dashed and solid lines, respectively. (c) Scatter plot comparing the photoluminescence mean and variance of theoretically calculated defect line shapes with the experimental data of emitter A. The most likely defect transitions are shown in the right panel which is the region close to the experimental data, marked by the square in the left panel. (d) Euclidean distances of all defect transitions in corresponding colors to (c) for emitter A, B and C. The histogram on the right shows the distances for the five most likely defect candidates. (e) Euclidean distances for emitter D to L in corresponding colors to (c). All emitters show small distances for the same five defect transitions. (f) Schematics of C_2_C_B_, C_2_C_N_, and V_N_C_B_ where the latter is taken from ref. 20. All experimental photoluminescence line shapes are background-corrected as outlined in ESI V.† We note that the PBE functional is used for all defect transitions (including C_2_C_B_, C_2_C_N_[*β*], and V_N_C_B_[*S* = 1]), except for the inset in (b) where the HSE06 functional is used.

Fig. 1a shows the photoluminescence of a group I centre at room and low temperature. Decreasing the temperature results in a narrower ZPL and reveals a detailed structure of the PSB. When comparing photoluminescence line shapes for different group I centres at low temperature, we find variations among the PSB line shapes as shown in ESI I.† These differences correspond to variations in the electron–phonon coupling of the defect transition among the luminescent centres, and may be due to different underlying defects. It is therefore necessary to compare several different defect transitions to individual luminescent centres, as outlined below. This is in contrast to comparing total Huang–Rhys factors of several defect transitions with experiments, which neglects the detailed information about the spectral structure of the PSB.

### Screening of defect transitions

To get insight into the microscopic origin of 2 eV luminescent centres in hBN, we study 26 different defect transitions. We focus on carbon-based defects as these have been experimentally demonstrated to be responsible for luminescent centers emitting around 2 eV.^6^ Our generation process can easily generate carbon-based defects by incorporating ubiquitous hydrocarbons from the annealing environment as well as by carbon impurities intrinsically present in the hBN.^7^ Furthermore, despite the use of oxygen atoms during irradiation, oxygen-based defects^24^ are less likely to occur due to subsequent annealing in nitrogen atmosphere.^7^ We note that we use the PBE functional for this screening of defect transitions while the HSE06 functional is used for a more accurate study of a selected subset of defect transitions.

To obtain the theoretical photoluminescence line shapes, we use *ab initio* methods to calculate the ZPL energies and partial Huang–Rhys factors of all studied defect transitions (see ESI II†). We emphasize that our calculations are done on monolayer hBN, while our experiments are done on multilayered hBN. However, adding more layers in our calculations will not significantly change the spectrum of our calculated defects since the displacement vector between the ground and excited state configurations lies within the plane and thus is orthogonal to out-of-plane phonons (see also Methods). Therefore, the out-of-plane phonons coming from adjacent layers should not influence significantly the calculated spectra.

With the Huang–Rhys factors at hand, we can construct the spectral density^25^1
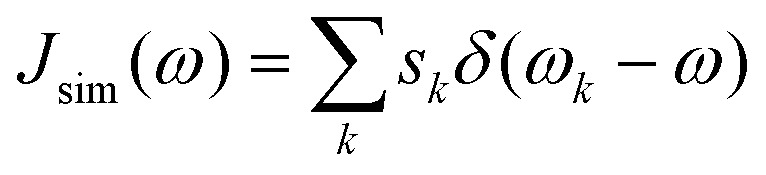


Here, *ω* is the angular frequency and *s*_*k*_ the partial Huang–Rhys factor associated to a phonon mode of angular frequency *ω*_*k*_. To account for the natural lifetime of phonons, we approximate the *δ*-functions with Gaussian functions as outlined in ESI III.† For details on the *ab initio* simulations, we refer the reader to the Methods.

With the spectral density at hand, we can calculate the dynamics and optical properties of the system using the polaron method. Here, the electronic states of the defect transition are dressed by vibrational modes of the phonon environment.^18,26^ This enables one to derive a quantum master equation that is non-perturbative in the electron–phonon coupling strength, and thus captures phonon sideband processes in the photoluminescence.^26,27^ In Fig. 1b we compare the photoluminescence of emitter A against the line shape for the carbon trimer C_2_C_B_, calculated using the polaron theory. The black bars show the partial Huang–Rhys factors (*s*_*k*_) illustrating the origin of the PSB from *ab initio* methods. We highlight that the line shape of C_2_C_B_ is unique for emitter A since we fit each theoretical line shape to individual experimental line shapes, with the ZPL linewidth as the only free fitting parameter (see ESI III†).

In order to screen the 26 different defect transitions calculated with *ab initio* methods, we calculate the photoluminescence mean *S* and variance *σ*, defined as,
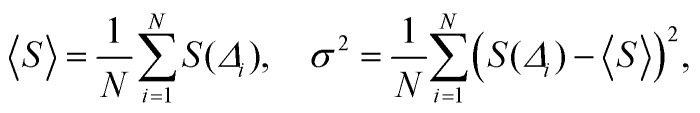
where *S*(*Δ*_*i*_) is the intensity of the spectrum at the ZPL detuning *Δ*_*i*_, and *N* is the number of data points (see Methods). If the photoluminescence mean and variance of a defect transition are close to the experimental values, the theoretical photoluminescence line shape is similar to the experimental one, thus providing a coarse approach to screening relevant defect transitions.

As an example, we apply this method to emitter A and plot the photoluminescence mean and variance for all calculated defect transitions along with the experimental values (Fig. 1c). Here, different colors correspond to different defects, some of which have several optically active transitions (details in ESI IV†). The scatter plot (in double logarithmic scale) shows that the photoluminescence mean and variance for most defect transitions differ significantly from the experimental data and thus are unlikely candidates, while the most likely defect transitions are clustered around the experimental data of emitter A, as shown in the right panel of Fig. 1c.

Inspired by work on Raman spectroscopy,^28^ we define an Euclidean distance *d* to the experiment
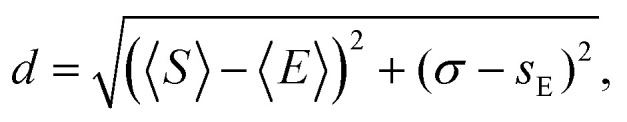
where, 〈*E*〉 and *s*_E_ are the experimental mean and variance while 〈*S*〉 and *σ* are the mean and variance for each defect transition. For emitter A, the line shape of C_2_C_B_ is very similar to the experiments, thus C_2_C_B_ is located closely to the experiment in the scatter plot (Fig. 1c) and shows a small Euclidean distance (Fig. 1d in logarithmic scale).

The scatter plot shown in Fig. 1c depends on the luminescent centre studied, since the ZPL line width differs among luminescent centres. Therefore, we calculate an individual scatter plot for each luminescent centre (for emitter B and C these are given in ESI IV†) with the resulting Euclidean distances shown along with emitter A in Fig. 1d. We find that the same five defect transitions show similar photoluminescence line shapes for emitter A, B and C. Expanding our analysis to twelve group I centres (line shapes shown in ESI I†), we find that for all experiments the five closest defect transitions are identical to the ones for emitter A (Fig. 1e). Therefore, we focus our attention on these five defect transitions while discarding the other 21 transitions.

### Second screening step

The statistical analysis described above is independent on the theoretical ZPL energy because we match the theoretical line shapes with the experimental ZPL energies. Thus, comparing theoretical with experimental ZPL energies can help to narrow down the microscopic origin even further. For twelve group I centres the experimental ZPL energies are between 2.0 and 2.3 eV (see Table 1) and thus theoretical ZPL energies much different to this range reveal less likely candidates. Among the five defect transitions showing small Euclidean distances to these twelve centres (Fig. 1), we neglect V_B_V_B_[*β*] and C_2B_V_N_^−1^[*S* = 1] since their predicted ZPL energies are too high and too low, respectively (see Table 1). Furthermore their total Huang–Rhys factors are too low, 0.3 and 0.6 (see ESI II†), respectively, to reflect the relatively strong electron–phonon coupling strength observed experimentally in the form of marked PSBs. On the contrary, the ZPL energies of C_2_C_B_, C_2_C_N_[*β*], and V_N_C_B_[*S* = 1] are similar to the experiments as shown in Table 1. In the following, we call these three defects C_2_C_B_, C_2_C_N_, and V_N_C_B_. The ZPL energies of these three defects show good agreement with experiments, while even the relatively more accurate HSE06 functional shows slightly smaller energies compared to the experimental values.

**Table 1:** Theoretical and experimental ZPL energies. The applied functional (PBE or HSE06) for the calculations of the defect transitions is given by the column name while the range of ZPL energies of emitter A to L are shown in the rightmost column (details in ESI I†). We did not use the HSE06 functional for V_B_V_B_[*β*] and C_2B_V_N_^−1^[*S* = 1] since their PBE results are very different to the experimental ZPL energies. The spin minority channel is labelled by *β* and *S* = 1 denotes the triplet state while we suppress the notations for spin majority channel and other spin states (details in ESI II†)

Defect transition	PBE	HSE06	Experiments
C_2_C_B_	1.16 eV	1.36 eV	2.0–2.3 eV
C_2_C_N_[*β*]	1.51 eV	1.67 eV	2.0–2.3 eV
V_N_C_B_[*S* = 1]	1.39 eV	1.75 eV	2.0–2.3 eV
V_B_V_B_[*β*]	2.70 eV	—	2.0–2.3 eV
C_2B_V_N_^−1^[*S* = 1]	0.90 eV	—	2.0–2.3 eV

Interestingly, the Euclidean distances for emitter A, B, and C (Fig. 1d) indicate that these emitters are of different microscopic nature. Indeed, the Euclidean distances, for the defect transitions C_2_C_B_ and C_2_C_N_, are the shortest for emitter A and B, respectively, while the defect transition V_N_C_B_ is the closest defect to emitter C. Added to the energy variations in the ZPLs between emitters A, B, and C (ESI I†) observed experimentally, we can infer that these three emitters have probably a different microscopic origin.

All in all, we can exclude 23 defect transitions for twelve group I centres and identified three most likely candidates: C_2_C_B_, C_2_C_N_, and V_N_C_B_. Fig. 1f shows the schematics of these three defect transitions. We note that V_N_C_B_ needs a nearby defect to populate the ground state, because the intersystem crossing from the singlet state can be neglected at our experimental detunings (see ESI VI†).

### PLE spectroscopy

In contrast to the generating function approach, our polaron formalism allows to introduce an external driving field in the system Hamiltonian, thereby directly simulating PLE experiments. For the latter, the photoluminescence is measured as a function of laser detuning which is defined as the laser energy minus the ZPL energy of the studied luminescent centre.

**Fig. 2:**
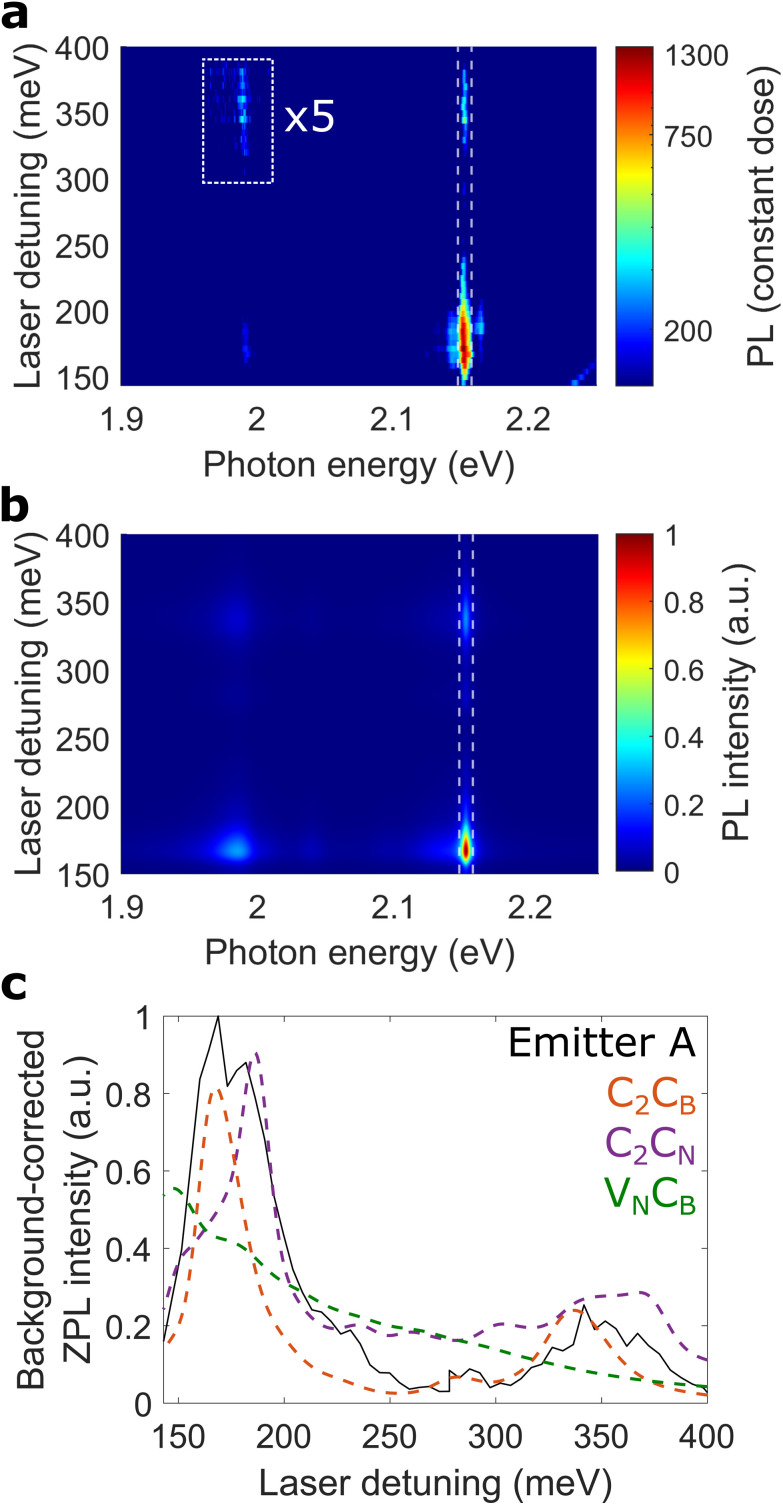
PLE spectroscopy. (a) Experimental PLE map of emitter A at 10 K, background corrected as outlined in ESI IX.† The photoluminescence (PL) in the highlighted box is multiplied by 5. The dim upward line in the bottom right corner corresponds to the silicon Raman ∼520 cm^−1^. (b) Theoretical PLE map of C_2_C_B_, shifted to the experimental ZPL energy of 2.153 eV. Here, the HSE06 functional is used and acoustic phonons are included in the spectral density. (c) Comparison of the experimental ZPL intensity (solid black line), indicated by white vertical dashed lines in (a) and (b), with C_2_C_B_ (orange dashed line), C_2_C_N_ (purple dashed line), and V_N_C_B_ (green dashed line). We note that the experimental ZPL intensity is rescaled to 170 meV and background-corrected as outlined in ESI IX.† Furthermore, all theoretical curves include acoustic phonons as described in the main text.

Fig. 2a shows the experimental PLE data (in logarithmic scale) of emitter A, *i.e.* the photoluminescence as a function of laser detuning (details and further group I centres in ESI VII and VIII†). The ZPL emission at photon energies around 2.15 eV is strong at a detuning of 168 meV and shows intermediate strength at 341 meV detuning. Moreover, the PSB emission around 1.99 eV is enhanced at the same laser detunings. In particular, the enhanced PSB emission at 341 meV detuning (highlighted by the white box) shows strong evidence of a phonon-assisted mechanism, as outlined below.

To model the PLE results precisely, we use the relatively more accurate HSE06 functional and study the photoluminescence line shape of emitter A in more detail. The experimental and theoretical line shape disagree at ZPL detunings from 10 to 50 meV, as shown by the inset of Fig. 1b. While this spectral range was previously associated with another, independent electronic transition,^29^ we assign this range to a low-energy, acoustic PSB from the same electronic transition since its PLE characteristic shows excellent qualitative agreement with the ZPL (see ESI IX†).

The experimentally observed acoustic PSB is not reproduced by our *ab initio* calculations, since the latter are carried out in a perfectly planar system with high symmetry that exhibits vanishing coupling to acoustic phonons, *i.e.* the corresponding partial Huang–Rhys factors are zero. This is in contrast to our experiments where the symmetry can be broken by local strain, non-radiative defects close by, non-parallel hBN layers as well as the vicinity of edges, kinks, and grain boundaries. Such a symmetry breaking allows the coupling to acoustic phonons which we observe in several experimental photoluminescence line shapes as asymmetric ZPL lines (see ESI I†). We highlight that the asymmetric ZPL observed in our experiments has been reported in several works on group I centres^6,7,30–33^ independent on the generation process.

To account for the aforementioned coupling to acoustic phonons, we modify the spectral density shown in eqn (1). We include acoustic phonons by adding a Gaussian contribution such that the total spectral density is written as *J*(*ω*) = *J*_sim_(*ω*) + *J*_acoustic_(*ω*) with*J*_acoustic_(*ω*) = *αω*_c_^−2^*ω* exp(−*ω*^2^/*ω*_c_^2^),where *α* and *ω*_c_ are found by fitting the photoluminescence spectrum. This form of spectral density is commonly used to describe the bulk acoustic phonons in three dimensions resulting from deformation potential coupling observed in semiconductor quantum dots.^18,34^ We find that the experimental photoluminescence at small ZPL detunings is closely resembled by calculations that include acoustic phonons, showing a very precise theoretical description (see inset of Fig. 1b). We refer the reader to the Methods section for more details on the theory and the fitting procedure used.

With the total spectral density at hand, we calculate the complete PLE spectrum of several defect transitions. While the commonly-used generating function approach is limited to the comparison of photoluminescence line shapes, our polaron-based method allows to directly calculate the theoretical PLE maps by introducing an external driving field into the system Hamiltonian. For each laser detuning we calculate the photoluminescence spectrum by sweeping out the experimental detuning range. We highlight that for the obtained PLE maps we use the phonons of the ground state, *i.e.* the identical phonons utilised for the emission line shape. To the best of our knowledge, this is the first time that PLE measurements of 2 eV luminescent centres in hBN are replicated by a theoretical model.

Fig. 2b shows the complete PLE map of C_2_C_B_ while the maps of C_2_C_N_ and V_N_C_B_ are shown in ESI III.† We find excellent agreement of the experimental PLE map with C_2_C_B_ and C_2_C_N_, reflecting a very good match of both emission line shape as well as PLE maps. The theoretical PLE of C_2_C_B_ shows a slightly stronger enhancement at 341 meV detuning compared to the experimental data. This may be due to other nonradiative decay channels like shelving states^22^ which are not considered in our model.

To compare our PLE experiments with several defect transitions, we study the ZPL intensity which is defined as the area under the ZPL (indicated by vertical lines in Fig. 2a and b). Fig. 2c shows excellent agreement of the experimental ZPL intensity with C_2_C_B_ and C_2_C_N_ while V_N_C_B_ shows poorer agreement, thus allowing us to exclude the latter defect for emitter A.

All in all, our excellent agreement of the complete experimental PLE with our polaron-based calculations for C_2_C_B_ and C_2_C_N_ is outstanding compared to previous work.^35^ This finding infers that, among the studied defect transitions, C_2_C_B_ and C_2_C_N_ are the most likely microscopic origins for emitter A. We highlight that the generation mechanism of emitter A, outlined in ref. 7, also holds for C_2_C_B_ and C_2_C_N_, since both defects can be formed by merging of C_N_ and C_B_ during thermal annealing.

Previously, experimental photoluminescence line shapes were compared with vacancies,^2,36^ oxygen-based defects^37^ and carbon-based defects.^6–8^ Although emitter A shows good agreement with C_2_C_B_ and C_2_C_N_, other luminescent centres, also generated by our process, show agreement with different defects. One example is emitter C (see Fig. 1d) showing a small Euclidean distance for V_N_C_B_, *i.e.* a good agreement with this defect. Furthermore, our irradiation-based process also generates luminescent centres with line shapes different to group I centres that show small Euclidean distances to defects different to C_2_C_B_, C_2_C_N_, and V_N_C_B_ (see ESI X†). We are convinced that our process generates several types of defects that appear as 2 eV luminescent centres in our experiments. Therefore, comparing PLE maps of individual luminescent centres with our polaron method helps to identify their microscopic origins.

### Excitation mechanism

The microscopic origin of twelve group I centres has been narrowed down to three defect transitions (Fig. 1). These three transitions have relatively large Huang–Rhys factors around 170 meV and thus can be modelled as two-level systems with discrete vibronic energy levels (see ESI II†). To verify the vibronic structure of these energy levels, which are directly related to the excitation mechanism, PLE is a strong tool since it probes off-resonant transitions in both ground and excited states.

The excitation mechanism of 2 eV luminescent centres in hBN does not involve interband transitions since the photon energy of the excitation laser (∼2.5 eV) is much smaller than the band gap *E*_g_ ∼ 6 eV.^38^ Supported by charge transfer experiments,^39^ the excitation mechanism most likely involves only electronic states inside the band gap, so-called deep levels. This makes the energy of the driving laser very important since only few, discrete electronic states are available – in contrast to a continuum of states for interband transitions in semiconductors.

**Fig. 3:**
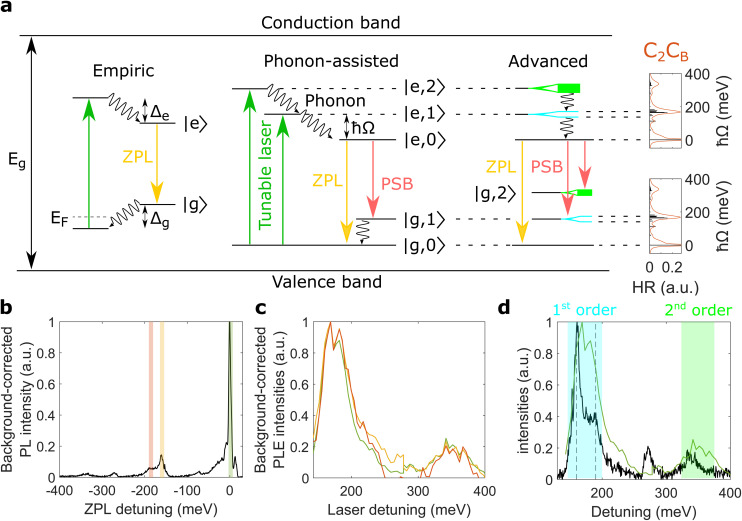
Excitation mechanism of emitter A. (a) Empiric, phonon-assisted, and advanced excitation mechanisms. The zero-phonon line (ZPL) and the phonon sidebands (PSB) are shown in yellow and red, respectively. The empiric mechanism has relaxation processes with energies *Δ*_e_ and *Δ*_g_ while the phonon-assisted mechanism shows relaxation *via* phonons with energy *ħΩ*. Here, the excited states are labelled with |e, *n*〉 corresponding to the electron in the excited state and its environment occupied by *n* phonons. The advanced mechanism is also phonon-assisted but with split first-order states |e, 1〉 and |g, 1〉. The rightmost panel shows the partial Huang–Rhys factors (HR) and the photoluminescence line shape for C_2_C_B_ in black and red, respectively. This photoluminescence line shape is obtained with the HSE06 functional and acoustic phonons. Further details are presented in the Methods. (b) The spectral ranges of two optical PSB are shaded in orange and yellow on top of the photoluminescence spectrum at 168 meV laser detuning. The bandwidth for the PSB intensities is 10 meV, identical to the one used for the ZPL intensity. The spectral range of the ZPL (green) is identical to the dashed lines in Fig. 2a. We assign the peak around 270 meV to another luminescent centre while the peak around 340 meV corresponds to the second-order PSB. (c) ZPL and PSB intensities in colors corresponding to the spectral range of integration shown in (b). We highlight that the ZPL intensity is identical to Fig. 2c. (d) ZPL intensity in green and flipped photoluminescence in black. The first- and second-order phonon states are shaded in colors corresponding to the advanced mechanism in (a). The vertical dashed lines are at detunings of 160 and 190 meV, respectively. We note that all photoluminescence (PL) and PLE intensities are background-corrected and normalised (see ESI V and IX†).

For 2 eV luminescent centres in hBN, one possible configuration of these deep levels is the empiric mechanism illustrated in Fig. 3a. Here, the driving laser excites an electron from a deep level below the Fermi level *E*_F_ to another level lying far below the conduction band. From here, the electron relaxes to the excited state |e〉 by dissipating energy *Δ*_e_. Then, a photon belonging to the ZPL is emitted by a transition to the ground state |g〉, followed by another relaxation with energy *Δ*_g_.

Recent PLE experiments^35,40^ observed an enhanced ZPL emission at detunings around 170 meV. While the authors assigned these results to a phonon-assisted process, the empiric mechanism can in principle also be the underlying excitation mechanism with *Δ*_e_ + *Δ*_g_ ∼ 170 meV. On the contrary, our PLE measurements show enhanced ZPL intensities both at detunings around 170 and 340 meV (see Fig. 2c). The empiric mechanism could be expanded by another electronic level, but the simplest explanation of our PLE results is the phonon-assisted excitation mechanism.

### Phonon-assisted mechanism

As shown above, our PLE measurements cannot be explained by the empiric mechanism. Therefore, we introduce the phonon-assisted excitation mechanism, also called Huang–Rhys model (see Fig. 3a) which was proposed in previous works on hBN.^35,41^ Here, two electronic states couple to one phonon mode with energy *ħΩ*, resulting in discrete vibronic energy levels |e, *n*〉 and |g, *m*〉. The driving laser excites a high laying vibronic state |e, *n*〉 in the excited electronic manifold which rapidly relaxes to its lowest state |e, 0〉. A photon is then emitted through an electronic transition to the ground-state manifold, producing ZPL and PSB emission.

By varying the photon energy of the driving laser, we can resonantly excite different vibronic states |e, *n*〉 in the excited-state manifold (see Fig. 3a). In particular, the ZPL and PSB emission are enhanced at equally-spaced laser detunings of *ħΩ* and 2*ħΩ*, also called one- and two-phonon detuning. We observe this in our PLE experiments by studying the ZPL intensity (see Fig. 2c) but also by studying two PSB intensities, as outlined below.

To study PSB emissions as a function of laser detuning, we define the PSB intensity as the area under the PSB, indicated by shaded areas in Fig. 3b. We find an excellent qualitative agreement of the ZPL intensity with the two PSB intensities that are taken at ZPL detunings of around 160 and 190 meV (see Fig. 3c). This finding shows that the ZPL and the two optical PSB have the same excitation mechanism and thus can be used to investigate the excitation mechanism of emitter A. To the best of our knowledge, this is the first time that ZPL and PSB intensities of 2 eV luminescent centres in hBN are directly compared with each other.

The ZPL and PSB intensities are enhanced at equally-spaced detunings of ∼170 and ∼340 meV, as shown in Fig. 3c. This experimental finding agrees excellently well for *ħΩ* ∼ 170 meV with the phonon-assisted mechanism where ZPL and PSB emission are enhanced at detunings of *ħΩ* and 2*ħΩ*. At detunings between the two aforementioned energies, we obtain small ZPL and PSB intensities because the excitation laser energy is between the two states |e, 1〉 and |e, 2〉. Furthermore, the photoluminescence (Fig. 3b) shows first- and second-order PSB at detunings around 170 and 340 meV corresponding to optical transitions from |e, 0〉 to |g, 1〉 and |g, 2〉, respectively. Therefore, our experiments allow us to discard the empiric mechanism for emitter A and confirm indications of a phonon-assisted mechanism for group I centres in hBN. This might pave the way towards a universal excitation mechanism, which could also explain other experimental findings such as high-temperature photoluminescence^42^ and photophysics.^22,43^

In the phonon-assisted excitation mechanism (see Fig. 3a), the ZPL emission *via* |e, 2〉 (two-phonon detuning) and the PSB emission *via* |e, 1〉 (one-phonon detuning) are both processes involving two phonons, and thus should show comparable intensities (details in Methods). Indeed, our experimental ZPL intensity at two-phonon detuning shows similar strength as both PSB intensities at one-phonon detuning, since both processes involve two phonons (see ESI IX†). Furthermore, the ZPL intensity *via* |e, 1〉 involves only one phonon and thus is stronger than both aforementioned two-phonon processes. These results further support the phonon-assisted excitation mechanisms of emitter A.

### Detailed phonon coupling

The experimental photoluminescence of emitter A shows a first-order PSB at detunings around 170 meV which consists of two distinct peaks at 160 and 190 meV, shown by dashed lines in Fig. 3d. Furthermore, we find that the ZPL intensity also shows a strong peak at detunings around 170 meV with two shallower peaks at 160 and 190 meV. This reveals that both first-order states |e, 1〉 and |g, 1〉 are split into two distinct levels, reflected in the advanced excitation mechanism (see Fig. 3a). Here, the second-order phonon replicas |e, 2〉 and |g, 2〉 are shown as continua since our experiments do not show distinct peaks at two-phonon detuning (see Fig. 3d). The calculated HR factors of C_2_C_B_ (Fig. 3a) as well as split first-order PSB of several group I centres (see ESI I†) support that several phonon modes are involved in the excitation mechanism of group I centres in hBN. We note that previous works have assigned a split |g, 1〉 to longitudinal and transverse optical bulk phonons.^15,21,44^ In our work, we provide a more accurate description since we calculate the phonon modes coupled to the defect in the lattice, thereby taking into account both localised and delocalised phonon modes.

In Fig. 3d we compare the ZPL intensity with the flipped photoluminescence line shape. For the 160 and peaks we observe a blueshift and a redshift between the photoluminescence and ZPL intensity, respectively. This could point towards different phonon coupling between ground and excited states. While the redshift of the 190 meV peak is in agreement with previous work,^40^ the blueshift for the 160 meV peak has not been observed. Both spectral shifts (around 10 meV) are, however, on the same order of magnitude as the spectral resolution of both PLE setups (around 5 meV) and thus further PLE experiments with higher spectral resolution are needed to confirm the presence of fine differences in the phonon coupling between ground and excited states.

## Conclusion

In summary, we have studied the photoluminescence of luminescent centres in hBN with a focus on group I centres showing pronounced PSB around 170 meV and a ZPL energy around 2 eV. By combining *ab initio* methods with the non-perturbative polaron method, we calculated the emission line shapes of 26 different defect transitions. Studying both the theoretical ZPL energy and the Euclidean distance between experiment and theory showed that C_2_C_B_, C_2_C_N_, and V_N_C_B_ are the most likely defect candidates for twelve experimental line shapes. Our method of using the Euclidean distance represents a new tool to narrow down the number of possible defects responsible for luminescent centres in insulators as well as to make accurate predictions of their optical properties when integrated in photonic structures.

Our method of analysis represents a complement to the common methodology, which compares theoretical values of the total Huang–Rhys or Debye–Waller factor with values extracted from the experiment. Indeed, in this work, variations in the calculated photoluminescence intensity *S*(*Δ*_*i*_) due to the finer structure in the PSB are taken into account through the variance *σ*. Capturing these fine details in the PSB is crucial to construct accurate models of the electron–phonon coupling dynamics. Indeed, large variations in the spectral density caused by spectrally sharp phonon modes can induce non-trivial phonon–electron–photon correlations.^25^

The full potential of our new approach, combining *ab initio* calculations with the polaron method, comes into light when compared to PLE measurements of our group I centers. In contrast to the generating function approach, our polaron model allows introducing an external driving field and thereby enables us to calculate theoretical PLE maps. By focusing on the most likely defect candidates and adding acoustic phonons to the spectral density, we found excellent agreement of the experimental PLE of one group I centre with C_2_C_B_ and C_2_C_N_ while we could exclude 24 other defect transitions. Our excellent agreement of experimental PLE with *ab initio* calculations is outstanding compared to previous work.^35^

In our PLE experiments, we observed enhanced ZPL and PSB emission at one- and two-phonon detunings, the latter for the first time to the best of our knowledge. By resolving first- and second-order phonon transitions, we confirmed indications of a phonon-assisted excitation mechanism with a phonon energy around 170 meV. In particular, it is very unlikely that other electronic states are equally-spaced by 170 meV for both ground and excited states. Moreover, such additional electronic states are not predicted by *ab initio* calculations for the most likely defect transitions. Our findings are supported by split first-order PSB of several group I centres as well as by a decent agreement of our excitation mechanism with *ab initio* calculations of C_2_C_B_. The methodology of combining PLE measurements and the polaron method with input from *ab initio* methods can be applied to identify the microscopic origin of other luminescent centres or single-photon emitters in hBN and other materials.

We are convinced that the presented comparison of experimental PLE maps with advanced models, able to combine atomistic calculations with open quantum system theory, provides the most accurate description of 2 eV luminescent centres in hBN and their excitation mechanism. For luminescent centres showing excellent agreement with C_2_C_B_ and C_2_C_N_, lifetime measurements can give further insight since the theoretical values differ significantly.^9^ To prove the quantum nature of the studied luminescent centres, auto-correlation (*g*^(2)^ function) and cross-correlation measurements^21^ as well as polarisation-dependent measurements^41,45^ are required. Furthermore, bleaching^23,32^ and blinking^7,43,46^ of 2 eV luminescent centres are unresolved challenges since only few centres were stable over minutes^2,7,21,42,47^ or even months.^31^ All in all, further experimental and theoretical work is needed to both unambiguously identify the nature of group I centres and to make 2 eV luminescent centres in hBN suitable for applications in quantum technologies.

As a last observation, we would like to point out that the ubiquitously used 532 nm laser efficiently excites luminescent centres with ZPL energies around 2.15 eV because the laser energy of 2.33 eV matches the one-phonon detuning of around 170 meV. This fortunate coincidence of the energy of an ubiquitously used laser with the one-phonon detuning has been a gift for the research field and explains why the majority of works on hBN report 2 eV luminescent centres.

## Methods

### Scatter plot

The mean and variance shown in Fig. 1c and ESI IV† are calculated for ZPL detunings from −440 to 10 meV. We note that the only fitting parameter is the ZPL line width while the phonon broadening is kept constant (see ESI III†).

The scatter plots reflect the agreement of the partial Huang–Rhys factors of each defect transition with the experimental line shape. The discussed scatter plots are similar to a recent theoretical study^12^ focusing on the combined defect and PSB emission spectrum (*i.e.* accessing the vibrational fine structure).

We note that in our scatter plots we focus on the mean and variance while higher-order variances need to be considered for perfect agreement between theory and experiment.

### *Ab initio* calculations

All the *ab initio* calculations for the ground states, excited states and normal modes were performed within the GPAW electronic structure code^48^ using a plane-wave basis set with 800 eV plane wave cut-off and a Γ-point sampling of the Brillouin zone. All the defects were represented in a 7 × 7 × 1 supercell (monolayer) and allowed to fully relax until the maximum force was below 0.01 eV Å^−1^. A vacuum of 15 Å was used in the vertical direction.

The PBE exchange correlation (xc-)functional^49^ was used for all calculations. In addition, for C_2_C_B_, C_2_C_N_, and V_N_C_B_ the relatively more accurate HSE06 functional was used for the calculation of the ZPL energies. The excited states were calculated using the direct optimisation-maximum overlap matrix (DO-MOM) method^50^ with a maximum step length, *ρ*_max_, for the quasi-Newton search direction of 0.2. Compared to the standard Δ-SCF approach, the DO-MOM method yields improved convergence and avoids variational collapses during the SCF optimisation.^50^ We highlight that the C_2_C_N_ described here is the transition studied in ref. 9. For further details on the theory and calculation details we refer to our previous work.^51^

In all calculations, we considered in-plane defect structures and verified their dynamical stability by the absence of imaginary frequencies in the Γ-point phonon spectrum. Only for the excited state of V_N_C_B_, *i.e.* (2)^3^*B*_1_, does the carbon atom of the defect relax out of plane. The out-of-plane configuration is stable compared to the planar configuration by 0.23 eV for monolayer hBN. This energy difference is only 0.036 eV in a trilayer hBN with a V_N_C_B_ defect embedded in the central hBN layer (of the trilayer hBN).^7^

For specific defects we have considered charge states 0, +1, −1 (see ESI II†). These defects were selected based on the presence of nearby in-gap states that can capture or release an electron to the optically active electronic states (HOMO–LUMO).

### Optical spectroscopy

The optical characterisation is described in detail in ESI XI.† For PLE measurements at low temperature, we used a supercontinuum white light laser in combination with a tunable laser line filter resulting in a PLE resolution of ∼5 meV.

### Open quantum system

To model experimental PLE maps we use information about the phonon modes extracted from the *ab initio* calculations to construct an open system model for the defect transition. The emitter is modelled as a two level system with ground and excited states |g〉 and |e〉 respectively, with transition energy *ħω*_e_. The emitter is driven by a continuous-wave laser with angular frequency *ω*_L_, and interacts both with vibrational and electromagnetic environments. The form of this interaction is given in the ESI XII.† To account for strong electron–phonon coupling and resultant PSB observed for luminescence centres, we make use of the polaron method.^18,52^ This approach dresses the system energy levels with modes of the vibrational environment using a unitary transformation, providing an optimised basis in which to do perturbation theory. We then derive a Born–Markov master equation in the transformed frame which is non-perturbative in the original electron phonon interactions. This allows us to describe the dynamics^52^ and, crucially, the PSB of the optical transition in question.^26,27^ For further details of the model described above, we refer the reader to the ESI III and XII.†

We note that the expression of *J*_acoustic_(*ω*) uses a different definition of the spectral density compared to ref. 18 which can be obtained by multiplying *J*_acoustic_ with *ω*^2^ and *α* with *ω*_c_^−2^. The physical description remains unchanged, *i.e.* in both cases a three-dimensional acoustic phonon environment is used.

### Excitation mechanism

In Fig. 3a, the band gap is given by *E*_g_ ∼ 6 eV (ref. 38) and the Fermi level is *E*_F_. Within the empiric mechanism, the two relaxation processes *Δ*_e_ and *Δ*_g_ are associated with |e〉 and |g〉, respectively. In the phonon-assisted and advanced excitation mechanisms, we do not show the Fermi level for clarity. Furthermore, the excited states are labelled with |e, *n*〉 corresponding to the electron in the excited state and its environment 160 occupied by *n* phonons. Similarly, |g, *m*〉 describes the electron in the ground state with *m* phonons in its environment.

### One- and two-phonon processes

To identify one- and two-phonon processes, we use the electronic states |e, *n*〉 and |g, *m*〉 of the phonon-assisted excitation mechanism presented in Fig. 3a.

At a laser detuning of ∼170 meV, we excite the electronic state |e, 1〉 that relaxes to |e, 0〉 by the emission of one phonon. From |e, 0〉, the ZPL emission is realised without any further phonon emission, while the PSB process involves another phonon that is emitted by the relaxation from |g, 1〉 to |g, 0〉. Therefore, the ZPL is a one-phonon process while the PSB is a two-phonon process. The above described two-phonon process holds for both optical PSB at and 190 meV as well as the acoustic PSB at ∼10 meV.

At laser detunings of ∼340 meV we excite the state |e, 2〉, introduced in the phonon-assisted mechanism in Fig. 3a. This state relaxes by the emission of two phonons to |e, 0〉 from where a ZPL emission is realised without any further phonon emission. Thus, this process is also a two-phonon process like the optical PSB at ∼170 meV detuning.

## Author contributions

M. F. and A. H. carried out the experiments. M. F. and N. S. analysed and interpreted the experimental data. A. S. designed and performed the *ab initio* calculations. K. S. T. supervised the *ab initio* calculations. J. I.-S. developed the open quantum systems model, and fitted the theoretical curves to the experimental data. M. F., A. S., J. I.-S., A. H., K. S. T., A. W. H. and N. S. discussed the experimental results and compared them with the theoretical predictions. A. S. and J. I.-S. equally contributed to the theoretical part of this manuscript. All authors contributed to the writing of the manuscript.

## Conflicts of interest

During the preparation of this manuscript we became aware about a thorough study of defects in hBN, focusing on tailoring the ZPL energy.^53^

## Supplementary Material

NR-015-D3NR01511D-s001

## References

[cit1] Aharonovich I., Englund D., Toth M. (2016). Solid-state single-photon emitters. Nat. Photonics.

[cit2] Tran T. T., Bray K., Ford M. J., Toth M., Aharonovich I. (2016). Quantum emission from hexagonal boron nitride monolayers. Nat. Nanotechnol..

[cit3] O'Brien J. L., Furusawa A., Vučković J. (2009). Photonic quantum technologies. Nat. Photonics.

[cit4] Al-Juboori A., Zeng H. Z. J., Nguyen M. A. P., Ai X., Laucht A., Solntsev A., Toth M., Malaney R., Aharonovich I. (2023). Quantum Key Distribution Using an Integrated Quantum Emitter in Hexagonal Boron Nitride. Adv. Quantum Technol..

[cit5] Degen C. L., Reinhard F., Cappellaro P. (2017). Quantum sensing. Rev. Mod. Phys..

[cit6] Mendelson N., Chugh D., Reimers J. R., Cheng T. S., Gottscholl A., Long H., Mellor C. J., Zettl A., Dyakonov V., Beton P. H., Novikov S. V., Jagadish C., Tan H. H., Ford M. J., Toth M., Bradac C., Aharonovich I. (2021). Identifying carbon as the source of visible single-photon emission from hexagonal boron nitride. Nat. Mater..

[cit7] Fischer M., Caridad J. M., Sajid A., Ghaderzadeh S., Ghorbani-Asl M., Gammelgaard L., Bøggild P., Thygesen K. S., Krasheninnikov A. V., Xiao S., Wubs M., Stenger N. (2021). Controlled generation of luminescent centers in hexagonal boron nitride by irradiation engineering. Sci. Adv..

[cit8] Jara C., Rauch T., Botti S., Marques M. A. L., Norambuena A., Coto R., Castellanos-Águila J. E., Maze J. R., Munoz F. (2021). First-Principles Identification of Single Photon Emitters Based on Carbon Clusters in Hexagonal Boron Nitride. J. Phys. Chem. A.

[cit9] Li K., Smart T. J., Ping Y. (2022). Carbon trimer as a 2 eV single-photon emitter candidate in hexagonal boron nitride: A first-principles study. Phys. Rev. Mater..

[cit10] Sajid A., Thygesen K. S. (2020). V_N_C_B_ defect as source of single photon emission from hexagonal boron nitride. 2D Mater..

[cit11] Tawfik S. A., Ali S., Fronzi M., Kianinia M., Tran T. T., Stampfl C., Aharonovich I., Toth M., Ford M. J. (2017). First-principles investigation of quantum emission from hBN defects. Nanoscale.

[cit12] Linderälv C., Wieczorek W., Erhart P. (2021). Vibrational signatures for the identification of single-photon emitters in hexagonal boron nitride. Phys. Rev. B.

[cit13] Auburger P., Gali A. (2021). Towards ab initio identification of paramagnetic substitutional carbon defects in hexagonal boron nitride acting as quantum bits. Phys. Rev. B.

[cit14] Schauffert H. , Stewart J. C., Ali S., Walser S., Hörner H., Prasad A. S., Babenko V., Fan Y., Eder D., Thygesen K. S., Hofmann S., Bayer B. C. and Skoff S. M., Characteristics of quantum emitters in hexagonal boron nitride suitable for integration with nanophotonic platforms, 2023

[cit15] Preuss J. A., Groll D., Schmidt R., Hahn T., Machnikowski P., Bratschitsch R., Kuhn T., de Vasconcellos S. M., Wigger D. (2022). Resonant and phonon-assisted ultrafast coherent control of a single hBN color center. Optica.

[cit16] Nguyen M., Kim S., Tran T. T., Xu Z.-Q., Kianinia M., Toth M., Aharonovich I. (2018). Nanoassembly of quantum emitters in hexagonal boron nitride and gold nanospheres. Nanoscale.

[cit17] Vogl T., Lecamwasam R., Buchler B. C., Lu Y., Lam P. K. (2019). Compact Cavity-Enhanced Single-Photon Generation with Hexagonal Boron Nitride. ACS Photonics.

[cit18] Nazir A., McCutcheon D. P. S. (2016). Modelling exciton–phonon interactions in optically driven quantum dots. J. Phys.: Condens. Matter.

[cit19] Golami O., Sharman K., Ghobadi R., Wein S. C., Zadeh-Haghighi H., da Rocha C. G., Salahub D. R., Simon C. (2022). *Ab initio* and group theoretical study of properties of a carbon trimer defect in hexagonal boron nitride. Phys. Rev. B.

[cit20] Sajid A., Reimers J. R., Ford M. J. (2018). Defect states in hexagonal boron nitride: Assignments of observed properties and prediction of properties relevant to quantum computation. Phys. Rev. B.

[cit21] Feldman M. A., Puretzky A., Lindsay L., Tucker E., Briggs D. P., Evans P. G., Haglund R. F., Lawrie B. J. (2019). Phonon-induced multicolor correlations in hBN single-photon emitters. Phys. Rev. B.

[cit22] Boll M. K., Radko I. P., Huck A., Andersen U. L. (2020). Photophysics of quantum emitters in hexagonal boron-nitride nano-flakes. Opt. Express.

[cit23] Martínez L. J., Pelini T., Waselowski V., Maze J. R., Gil B., Cassabois G., Jacques V. (2016). Efficient single photon emission from a high-purity hexagonal boron nitride crystal. Phys. Rev. B.

[cit24] Li S., Gali A. (2022). Identification of an Oxygen Defect in Hexagonal Boron Nitride. J. Phys. Chem. Lett..

[cit25] Svendsen M. K. , Ali S., Stenger N., Thygesen K. S. and Iles-Smith J., Signatures of Non-Markovianity in Cavity-QED with Color Centers in 2D Materials, 2022, https://arxiv.org/abs/2207.10630

[cit26] Iles-Smith J., McCutcheon D. P., Nazir A., Mørk J. (2017). Phonon scattering inhibits simultaneous near-unity efficiency and indistinguishability in semiconductor single-photon sources. Nat. Photonics.

[cit27] Iles-Smith J., McCutcheon D. P., Mørk J., Nazir A. (2017). Limits to coherent scattering and photon coalescence from solid-state quantum emitters. Phys. Rev. B.

[cit28] Taghizadeh A., Leffers U., Pedersen T. G., Thygesen K. S. (2020). A library of ab initio Raman spectra for automated identification of 2D materials. Nat. Commun..

[cit29] Bommer A., Becher C. (2019). New insights into nonclassical light emission from defects in multi-layer hexagonal boron nitride. Nanophotonics.

[cit30] Tran T. T., Elbadawi C., Totonjian D., Lobo C. J., Grosso G., Moon H., Englund D. R., Ford M. J., Aharonovich I., Toth M. (2016). Robust Multicolor Single Photon Emission from Point Defects in Hexagonal Boron Nitride. ACS Nano.

[cit31] Vogl T., Campbell G., Buchler B. C., Lu Y., Lam P. K. (2018). Fabrication and Deterministic Transfer of High-Quality Quantum Emitters in Hexagonal Boron Nitride. ACS Photonics.

[cit32] Mendelson N., Xu Z.-Q., Tran T. T., Kianinia M., Scott J., Bradac C., Aharonovich I., Toth M. (2019). Engineering and Tuning of Quantum Emitters in Few-Layer Hexagonal Boron Nitride. ACS Nano.

[cit33] Wigger D., Schmidt R., Del Pozo-Zamudio O., Preuß J. A., Tonndorf P., Schneider R., Steeger P., Kern J., Khodaei Y., Sperling J., de Vasconcellos S. M., Bratschitsch R., Kuhn T. (2019). Phonon-assisted emission and absorption of individual color centers in hexagonal boron nitride. 2D Mater..

[cit34] Krummheuer B., Axt V. M., Kuhn T. (2002). Theory of pure dephasing and the resulting absorption line shape in semiconductor quantum dots. Phys. Rev. B: Condens. Matter Mater. Phys..

[cit35] Grosso G., Moon H., Ciccarino C. J., Flick J., Mendelson N., Mennel L., Toth M., Aharonovich I., Narang P., Englund D. R. (2020). Low-Temperature Electron–Phonon Interaction of Quantum Emitters in Hexagonal Boron Nitride. ACS Photonics.

[cit36] Li X., Shepard G. D., Cupo A., Camporeale N., Shayan K., Luo Y., Meunier V., Strauf S. (2017). Nonmagnetic Quantum Emitters in Boron Nitride with Ultranarrow and Sideband-Free Emission Spectra. ACS Nano.

[cit37] Xu Z.-Q., Elbadawi C., Tran T. T., Kianinia M., Li X., Liu D., Hoffman T. B., Nguyen M., Kim S., Edgar J. H., Wu X., Song L., Ali S., Ford M., Toth M., Aharonovich I. (2018). Single photon emission from plasma treated 2D hexagonal boron nitride. Nanoscale.

[cit38] Cassabois G., Valvin P., Gil B. (2016). Hexagonal boron nitride is an indirect bandgap semiconductor. Nat. Photonics.

[cit39] Xu Z.-Q., Mendelson N., Scott J. A., Li C., Abidi I. H., Liu H., Luo Z., Aharonovich I., Toth M. (2020). Charge and energy transfer of quantum emitters in 2D heterostructures. 2D Mater..

[cit40] Malein R. N. E., Khatri P., Ramsay A. J., Luxmoore I. J. (2021). Stimulated Emission Depletion Spectroscopy of Color Centers in Hexagonal Boron Nitride. ACS Photonics.

[cit41] Jungwirth N. R., Fuchs G. D. (2017). Optical Absorption and Emission Mechanisms of Single Defects in Hexagonal Boron Nitride. Phys. Rev. Lett..

[cit42] Kianinia M., Regan B., Tawfik S. A., Tran T. T., Ford M. J., Aharonovich I., Toth M. (2017). Robust Solid-State Quantum System Operating at 800 K. ACS Photonics.

[cit43] Tran T. T., Zachreson C., Berhane A. M., Bray K., Sandstrom R. G., Li L. H., Taniguchi T., Watanabe K., Aharonovich I., Toth M. (2016). Quantum Emission from Defects in Single-Crystalline Hexagonal Boron Nitride. Phys. Rev. Appl..

[cit44] Khatri P., Luxmoore I. J., Ramsay A. J. (2019). Phonon sidebands of color centers in hexagonal boron nitride. Phys. Rev. B.

[cit45] Kumar A. , Samaner Ç., Cholsuk C., Matthes T., Paçal S., Oyun Y., Zand A., Chapman R. J., Saerens G., Grange R., Suwanna S., Ateş S. and Vogl T., Polarization dynamics of solid-state quantum emitters, 2023 10.1021/acsnano.3c08940 PMC10883057 38335970

[cit46] Li C., Mendelson N., Ritika R., Chen Y., Xu Z.-Q., Toth M., Aharonovich I. (2021). Scalable and Deterministic Fabrication of Quantum Emitter Arrays from Hexagonal Boron Nitride. Nano Lett..

[cit47] Xu X., Martin Z. O., Sychev D., Lagutchev A. S., Chen Y. P., Taniguchi T., Watanabe K., Shalaev V. M., Boltasseva A. (2021). Creating Quantum Emitters in Hexagonal Boron Nitride Deterministically on Chip-Compatible Substrates. Nano Lett..

[cit48] Enkovaara J., Rostgaard C., Mortensen J. J., Chen J., Dułak M., Ferrighi L., Gavnholt J., Glinsvad C., Haikola V., Hansen H. (2010). *et al.*, Electronic Structure Calculations with GPAW: a Real-Space Implementation of the Projector Augmented-Wave Method. J. Phys.: Condens. Matter.

[cit49] Perdew J. P., Burke K., Ernzerhof M. (1996). Generalized Gradient Approximation Made Simple. Phys. Rev. Lett..

[cit50] Levi G., Ivanov A. V., Jónsson H. (2020). Variational calculations of excited states via direct optimization of the orbitals in DFT. Faraday Discuss..

[cit51] Bertoldo F., Ali S., Manti S., Thygesen K. S. (2022). Quantum point defects in 2D materials - the QPOD database. npj Comput. Mater..

[cit52] McCutcheon D. P., Nazir A. (2010). Quantum dot Rabi rotations beyond the weak exciton–phonon coupling regime. New J. Phys..

[cit53] Cholsuk C., Suwanna S., Vogl T. (2022). Tailoring the Emission Wavelength of Color Centers in Hexagonal Boron Nitride for Quantum Applications. Nanomaterials.

