# Supplementary material for: Combining experiments on luminescent centres in hexagonal boron nitride with the polaron model and ab initio methods towards the identification of their microscopic origin
Source: Nanoscale. 2023 Aug 16;15(34):14215–26. doi: 10.1039/d3nr01511d (PMC10472209; doi:10.1039/d3nr01511d)
Supplement: NR-015-D3NR01511D-s001 [file NR-015-D3NR01511D-s001.pdf]

Electronic Supplementary Material (ESI) for Nanoscale.
This journal is © The Royal Society of Chemistry 2023

Supplementary Materials
Combining experiments on luminescent centres in hexagonal boron nitride with the polaron model and ab initio methods towards the identification of their microscopic origin

Moritz Fischer,[1,2,3] Ali Sajid,[2,4] Jake Iles-Smith,[5] Alexander Hötger,[6] Denys I. Miakota,[1] Mark K. Svendsen,[4] Christoph Kastl,[6] Stela Canulescu,[1] Sanshui Xiao,[1,2,3] Martijn Wubs,[1,2,3] Kristian S. Thygesen,[2,4] Alexander W. Holleitner,[6] and Nicolas Stenger[1,2,3,*]

[1]*Department of Electrical and Photonics Engineering, Technical University of Denmark, 2800 Kgs. Lyngby, Denmark*
[2]*Centre for Nanostructured Graphene, Technical University of Denmark, 2800 Kgs. Lyngby, Denmark*
[3]*NanoPhoton - Center for Nanophotonics, Technical University of Denmark, 2800 Kgs. Lyngby, Denmark*
[4]*Department of Physics, Technical University of Denmark, 2800 Kgs. Lynby, Denmark*
[5]*Department of Electrical and Electronic Engineering, The University of Manchester, Sackville Street Building, Manchester M1 3BB, United Kingdom*
[6]*Walter Schottky Institute and Physics Department, Technical University of Munich, 85748 Garching, Germany*

[*] Corresponding author. Email: niste@dtu.dk

I. FURTHER GROUP I SPECTRA

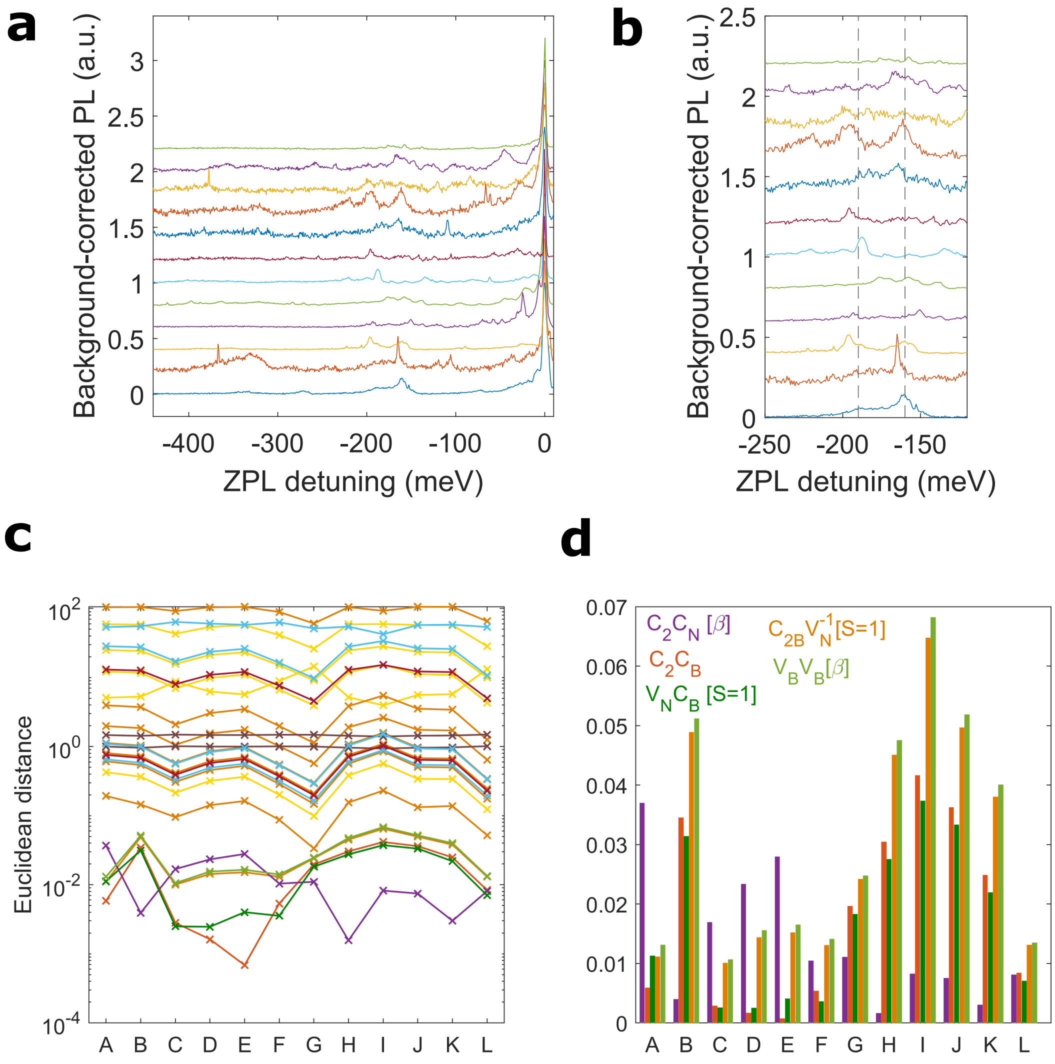

FIG. S1. **Low-temperature photoluminescence of luminescent centres belonging to group I.** (a) Photoluminescence line shapes at $T = 10\,\mathrm{K}$ under $2.37\,\mathrm{eV}$ excitation, shifted vertically for clarity. The spectra from bottom to top correspond to Emitter A to Emitter L, given by the x axis in (c). (b) Optical PSB of the luminescent centres shown in (a) with corresponding colors, shifted vertically for clarity. The vertical dashed lines are at detunings of 160 and 190 meV, respectively. (c) Euclidean distance for Emitter A up to Emitter L, with corresponding spectra shown in (a) from bottom to top. (d) Histograms of Emitter A to Emitter L. The colors correspond to the defect transitions given by the legend. All line shapes are background corrected as outlined in Supplementary Information V and more details are given in the main text.

A	B	C	D	E	F	G	H	I	J	K	L
2.1528	2.1785	2.0122	2.2500	2.1797	2.2045	2.2133	2.1652	2.2058	2.0819	2.1703	2.1984

TABLE S1. Zero-phonon line energies (in electron-volt) of Emitter A to L.

II. DETAILS ON *AB INITIO* CALCULATIONS

Nomenclature of defect transitions

In the main text, we suppress the notations for the spin majority channel α as well as non-triplet spin states. For C_2C_B, this results in

$$C_2C_B[S = 1/2, \alpha] \equiv C_2C_B \ , \quad (1)$$

where $\equiv$ stands for an equal representation. Another example is $C_2C_N[S = 1/2, \beta] \equiv C_2C_N[\beta]$.

		α			β		
Defect	Charge and spin state	ZPL(eV)	ΔQ	HR	ZPL(eV)	ΔQ	HR
$C_2C_N - V_N$	$C_2C_N - V_N[S = 1]$(E=2.15eV)	0.2	1.3	7.45	0.96	1.76	19.7
	$C_2C_N - V_N[S = -1/2]$(E=0.0eV)	1.96	0.8	5.72	–	–	–
	$C_2C_N - V_N^{+1}[S = 1/2]$	–	–	–	0.72	1.42	15.7
	$C_2C_N - V_N^{-1}[S = 1/2]$	–	–	–	1.96	1.3	9.1
$C_{2N}V_N$	$C_{2N}V_N[S = 1/2]$	2.08	1.083	7.16	–	–	–
	$C_{2N}V_N^{+1}[S = -1/2]$(E=0.0eV)	2.2	0.83	4.14	–	–	–
	$C_{2N}V_N^{-1}[S = 1]$(E=1.43eV)	1.7	0.72	3.82	1.07	0.41	1.29
	$C_{2N}V_N^{-1}[S = -1/2]$(E=0.0eV)	1.47	1.31	9.05	–	–	–
$C_{2B}V_N$	$C_{2B}V_N[S = 1/2]$	1.41	1.08	5.67	1.1	0.8	5.07
	$C_{2B}V_N^{-1}[S = -1/2]$	0.77	0.61	3.2	–	–	–
	$C_{2B}V_N^{-1}[S = 1]$(E=0.64eV)	0.9	0.29	0.635	1.17	0.8	4.99
	$C_{2B}V_N^{+1}[S = -1/2]$(E=0.0eV)	0.7	0.9	5.56	–	–	–
	$C_{2B}V_N^{+1}[S = 1]$(E=0.64eV)	1.12	1.39	9.91	0.74	1.19	10.8
$C_2C_B - V_B$	$C_2C_B - V_B[S = 1]$(E=0.0eV)	1.39	3.05	45	–	–	–
	$C_2C_B - V_B^{-1}[S = 1/2]$	0.74	13.52	36.8	–	–	–
V_BV_B	$V_BV_B[S = 1]$	–	–	–	2.7	0.41	0.3
$C_BC_NV_N$	$C_BC_NV_N^{+1}[S = 1]$(E=3.92eV)	1.37	0.92	10.5	–	–	–
	$C_BC_NV_N^{+1}[S = -1/2]$(E=0.0eV)	2.95	1.19	7.45	–	–	–
C_2C_N	$C_2C_N[S = 1/2]$	–	–	–	1.51	0.25	1.2
C_2C_B	$C_2C_B[S = 1/2]$	1.16	0.24	1.1	–	–	–
V_NC_B	$V_NC_B[S = 1]$	1.39	0.53	1.58	–	–	–
C_2C_N, HSE06	$C_2C_N[S = 1/2]$	–	–	–	1.67	0.26	1.26
C_2C_B, HSE06	$C_2C_B[S = 1/2]$	1.36	0.26	1.2	–	–	–
V_NC_B, HSE06	$V_NC_B[S = 1]$	1.75	0.53	1.50	–	–	–

TABLE S2. **Zero-phonon lines (ZPL), momentum displacements (ΔQ) and Huang-Rhys factors (HR) of all studied defects.** Here, α refers to the majority spin channel and β to the minority spin channel. The energy E in the second column gives the energy difference between the singlet and triplet states. It is only given for defects that show both a singlet and a triplet state. The PBE functional is used for all calculations except for the last three rows where the HSE06 functional was used.

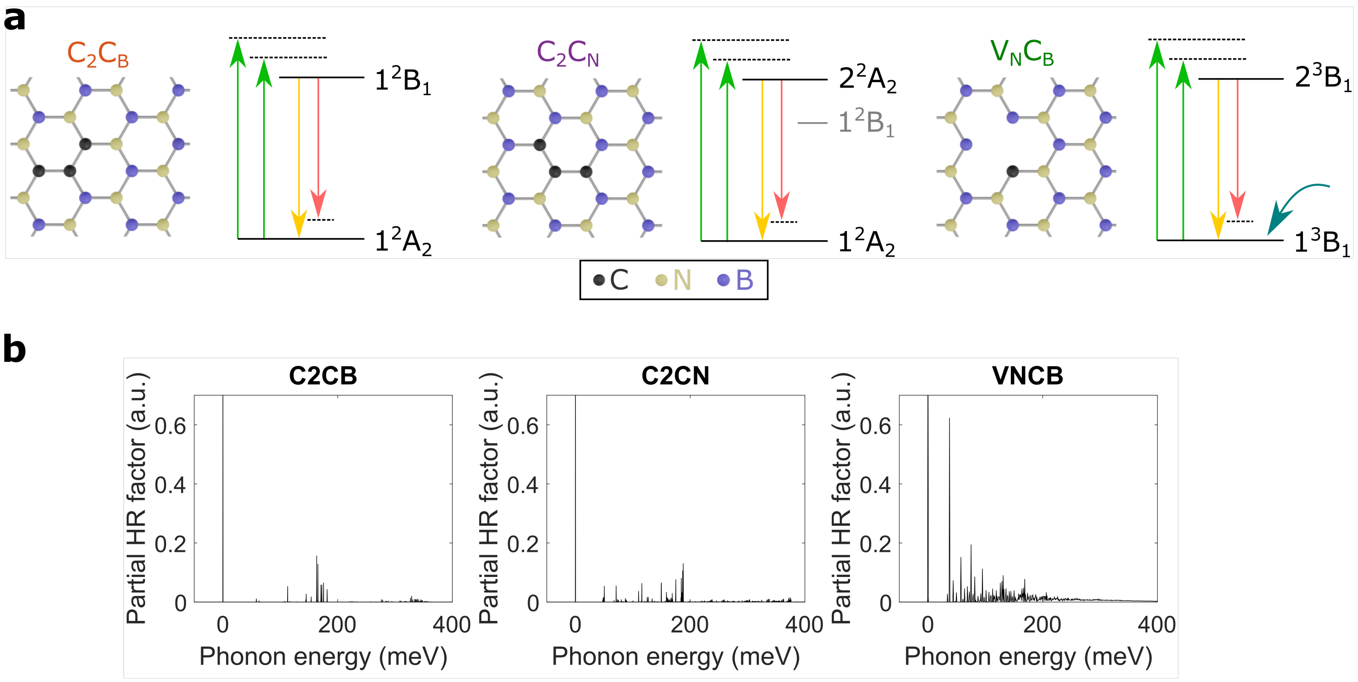

FIG. S2. **Schematics, electronic levels and partial Huang-Rhys factors for C_2C_B, $C_2C_N[\beta]$, and $V_NC_B[S = 1]$.** (a) Schematics and electronic levels of C_2C_B, $C_2C_N[\beta]$, and $V_NC_B[S = 1]$ where the latter is taken from Ref. [1]. (b) The partial Huang-Rhys factors for C_2C_B, $C_2C_N[\beta]$, and $V_NC_B[S = 1]$ are shown from left to right. We highlight that the HSE06 functional was used for all three defects.

III. FITTING AND COMPARISON WITH EXPERIMENT

In this section we outline the fitting procedure used to compare the polaron method to experiment. The fitting is done in two parts, in the first we extract the width of ZPL, the second fits the inhomogeneous broadening about the ZPL associated to acoustic phonons. To do both of these fits, we consider the emission lineshape driven by a excitation laser with detuning $\tilde{\Delta} = 168$ meV. We also assume that the phonons decay on a much faster timescale than light emission occurs, which allows us to ignore the excitation effects during the fitting procedure. This allows us to disregard the coherent driving term $\Omega_{\rm R}\sigma_x/2$ and the phonon dissipator $\mathcal{K}_{\rm PH}$ in Eq. 10, and assume that the emitter is initially in its excited state.

A. Fitting the zero-phonon line

To fit the ZPL, we restrict the experimental data to a frequency window ± 0.008 eV around the measured ZPL. This allows us to ignore the phonon sideband contribution, fitting only the ZPL with only function $S_{\rm opt}(\omega)$. For an emitter initially in its excited state, the ZPL spectrum reduces to a simple Lorentzian lineshape:

$$S_{\rm opt}(\Delta\omega) \approx \frac{B^2}{2\Gamma}\frac{\Gamma+\gamma}{(\Gamma+\gamma)^2+\Delta\omega^2}, \tag{2}$$

where $\Delta\omega$ is the detuning from the emitter frequency. After first normalising the data and the above expression to the peak of the ZPL, we do a least min-squared fit. Note that we can fit only a single rate, since Γ and γ combine to give a single width of the Lorentzian, thus we set $\gamma = 0$ without loss of generality. We find an optimal $\Gamma = 3.15$ meV with residual of $r = 0.098$ for Emitter A.

B. Describing electron-phonon sidebands in photoluminescence

As discussed in the main tex, we divide the phonon spectral density into two components $J_{\rm Ph}(\omega) = J_{\rm FP}(\omega) + J_{\rm A}(\omega)$. The first contribution, $J_{\rm FP}(\omega) = \sum_{\mathbf{k}} S_{\mathbf{k}}\delta(\omega-\omega_{\mathbf{k}})$, captures the phonons calculated from first principles, where $S_{\mathbf{k}}$ is the partial Huang-Rhys factor associated with a phonon with frequency $\omega_{\mathbf{k}}$ [2]. To move to a continuum limit version, we account for the natural lifetime of phonons in a material by approximating the δ-functions in the definition of $\mathcal{J}_{\rm Ph}(\nu)$ with a Gaussian function $\delta(\omega) \approx [\sigma\sqrt{2\pi}]^{-1}\exp[-(\omega/\sqrt{2}\sigma)^2]$, where σ is the phonon broadening parameter. We choose the broadening parameter as $\sigma = 5$ meV, such that it phenomenologically reproduces features in the emission spectrum at low temperature.

The second contribution to the phonon spectral density, $J_{\rm A}(\omega)$, is associated to acoustic phonons, and leads to the inhomogeneous broadening around the ZPL, which is not captured by the first-principle calculations. We assume that this spectral density takes the form $J_{\rm A}(\omega) = \alpha\omega_c^{-2}\omega e^{-\omega^2/\omega_c^2}$ [3], where α and ω_c are left as fitting parameters. This form of spectral density is commonly used in the semiconductor quantum dot literature, where it describes bulk acoustic phonons. Similarly to the ZPL fits we restrict ourselves to a frequency window of -0.06 eV $< \Delta\omega < 0.01$ eV. In contrast to the ZPL fits, however, we use the full expression for the emission spectrum, including the full phonon sideband given in Section XII C. The value of the optimal phonon parameters extracted from these fits changes depending on the defect. It was not possible to obtain a fit for the acoustic phonon sideband for V_NC_B due to the discrepancy between the sideband produced by first principles calculations and the measured PL. For illustrative reasons, we use the acoustic phonon parameters associated to C_2C_N when plotting the theoretical V_NC_B PL.

Fig. S3 shows the fitted PL lineshapes for the three defects considered in the manuscript. We compare the cases where acoustic phonons are neglected (dashed) and included (solid). For the C_2C_N and C_2C_B defect complexes, we see improved agreement about the ZPL when acoustic phonons are included. V_NC_B remains a are poor fit, with and without acoustic phonons. Notably, when acoustic phonons are included, we observe additional acoustic sidebands on the phonon replicas found from DFT calculations.

C. Theoretical photoluminescence emission spectroscopy

The model presented above, and the resulting fits, allow us to calculate the PLE directly from the full phonon model. We do this by varying the detuning of the continuous wave driving laser, Δ, and calculating the emission

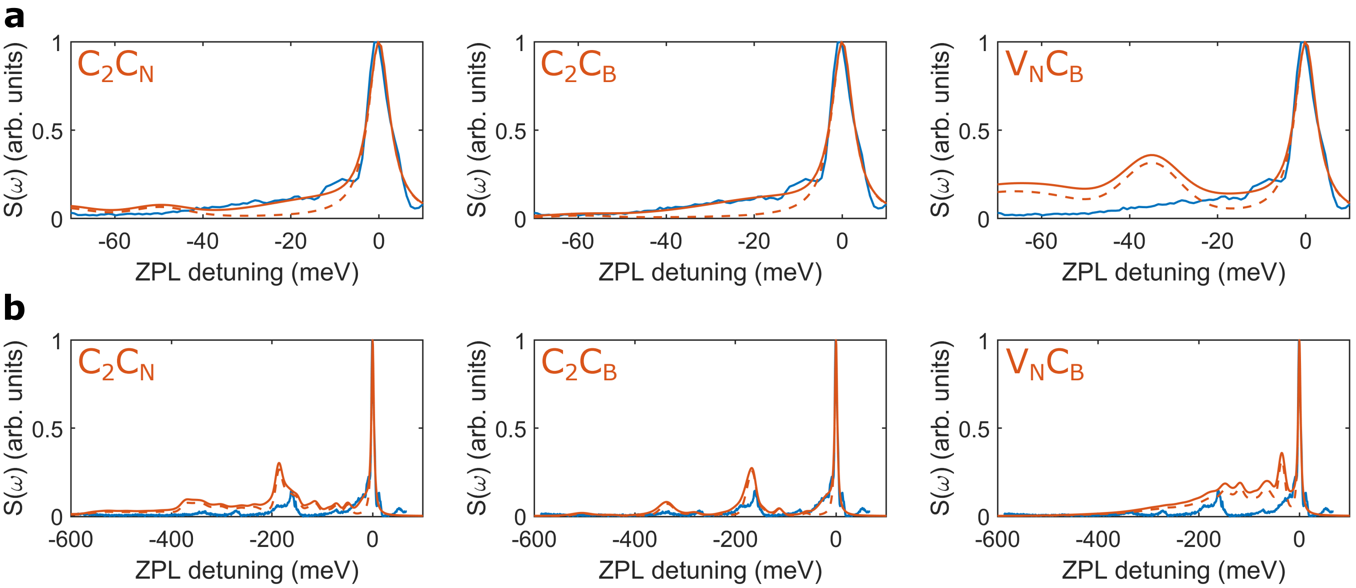

FIG. S3. **Comparison of the experiment (blue) and theory fits for three main defects considered in the main text.** The dashed curves include only phonons calculated through *ab initio* calculations, while the solid orange curves show the fits with acoustic phonons. The spectra plotted in (b,d,f) are the same as (a,c,e) respectively, plotted over a larger range. Temperature is set to $T = 10$ K.

spectrum for each detuning. For the driven case, we consider the steady-state emission spectrum, defined as:

$$S(\omega) = \mathrm{Re}\left[\lim_{t\to\infty}\int_0^\infty d\tau \mathcal{G}(\tau) g^{(1)}_{\mathrm{opt}}(t,\tau)\right], \tag{3}$$

where the integral over t has been reduced to the steady state limit.

Fig. S4 shows the PLE for the three defects considered in the main manuscript.

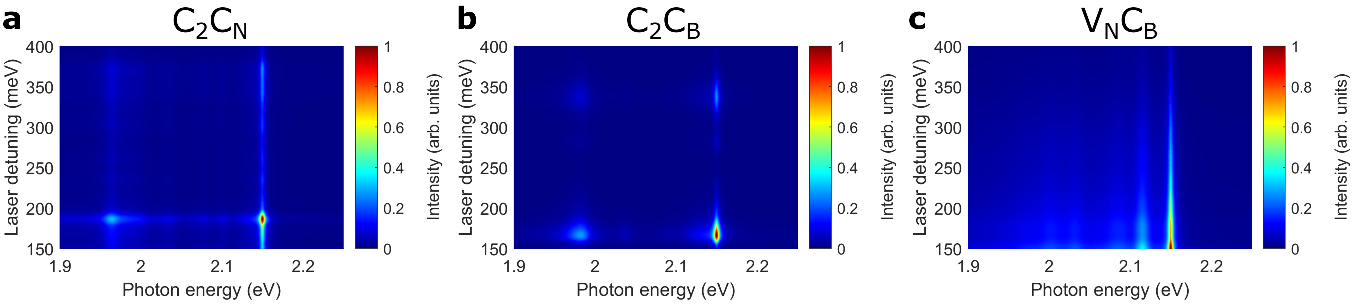

FIG. S4. **Theoretical PLE maps fitted to Emitter A.** The calculated PLE maps include electron-phonon interactions as described in the main text.

Fig. S5 shows the photoluminescence for $C_{2B}V_N^{-1}[S=1]$ which does not conform nicely with the experiment.

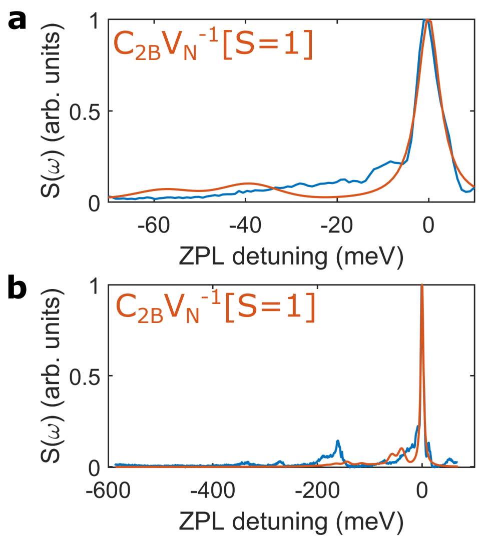

FIG. S5. **Comparison of the experimental photoluminescence (blue) with the theoretical spectrum of** $C_{2B}V_N^{-1}[S=1]$ **(red).** The theoretical spectrum is calculated without acoustic phonons. The spectra in (a) and (b) are identical but plotted over different ranges.

IV. DETAILED SCATTER PLOTS

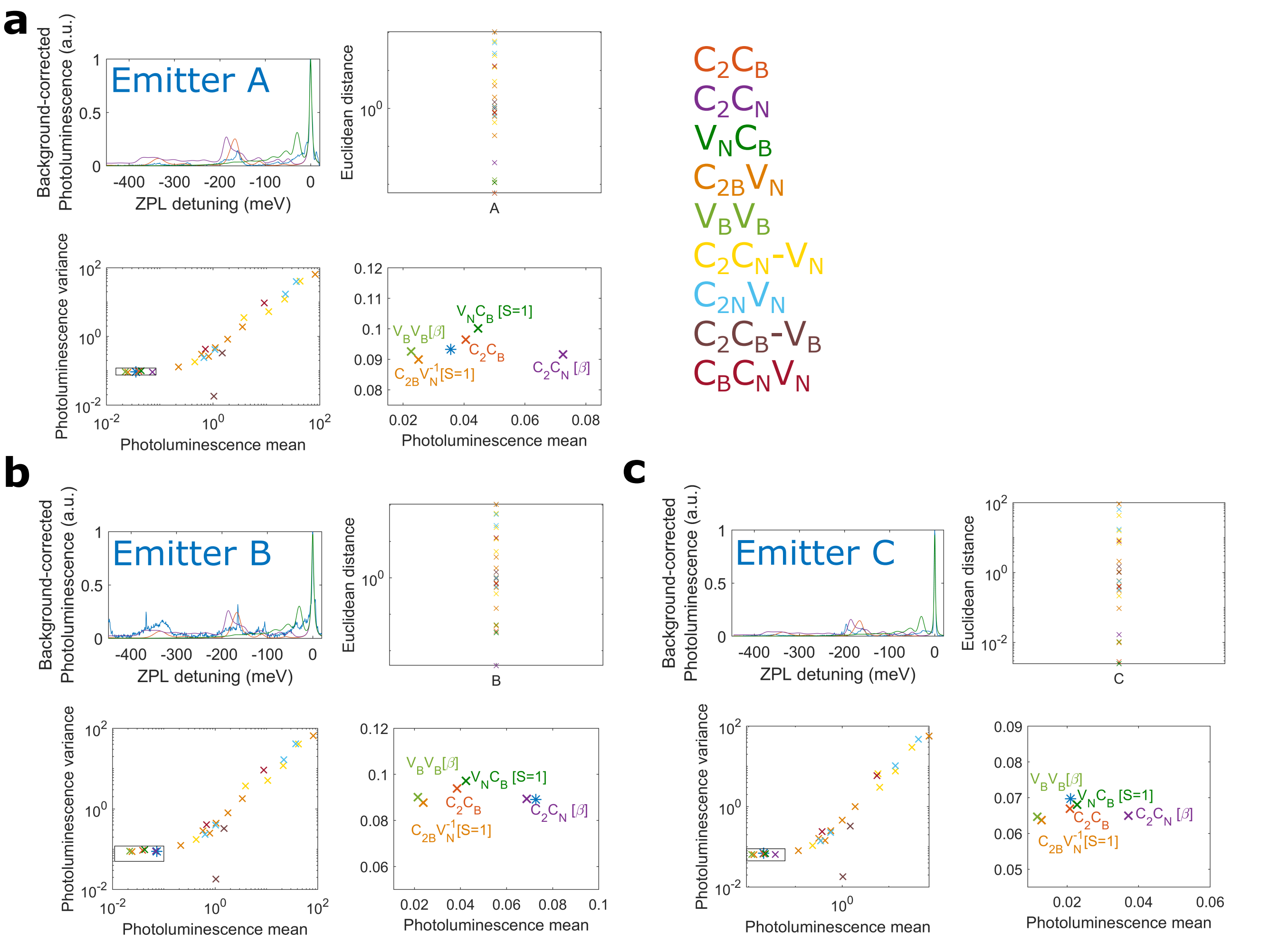

FIG. S6. **Detailed scatter plot for all studied defects.** (a) Emitter A. The top left panel shows the experimental photoluminescence as well as the theoretical line shapes of $C_2C_B[\beta]$ (red), C_2C_N (purple), and $V_NC_N[S=1]$ (dark green). The bottom panels show the scatter plot with a zoom-in on the right side, indicated by a square in the left plot. The top right panel shows the euclidean distance. The colors of the defects are given on the right. (b) Emitter B, labelling as in (a). (c) Emitter C, labelling as in (a).

V. BACKGROUND CORRECTION AND FURTHER LOW-TEMPERATURE PSB

For easy comparison of PSB, we carried out a background correction via a publicly available script [4]. We use this script with the asymmetric Huber function at fourth order and $s = 0$. In Fig. S7, we show the background correction for one photoluminescence spectrum.

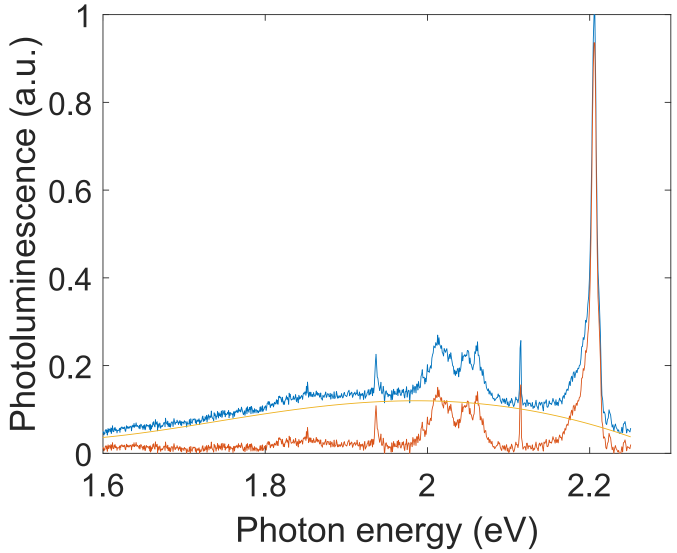

FIG. S7. **Background correction of photoluminescence line shapes.** Background correction of one photoluminescence spectrum. The raw spectrum is shown in blue, the background in yellow and the background-corrected spectrum in red.

VI. EXTENDED LEVEL MECHANISM OF V_NC_B

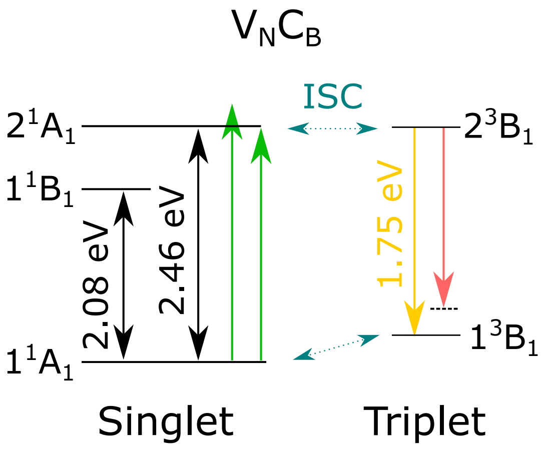

FIG. S8. **Extended level mechanism of V_NC_B.** The singlet system is shown on the left and the triplet system on the right. Emission from the triplet state is not possible by direct excitation because the ground state of the triplet state is not populated in equilibrium. However, optical excitation of the singlet system followed by an intersystem crossing (ISC) is possible. This requires laser detunings that are higher than $2.46 - 1.75\,\text{eV} = 0.71\,\text{eV}$. This is significantly larger than our experimental laser detunings from ~ 150 to $400\,\text{meV}$.

VII. FURTHER EXPERIMENTAL PLE DATA

Fig. S9 and S10 show the PLE data for Emitter A, B and C without any background correction, i.e. both photoluminescence and PLE intensities are shown without background correction.

Fig. S9a shows the photoluminescence spectra for several laser detunings. We obtain strong variations with laser detuning that are investigated in detail under continuous laser detuning, as shown in Fig. S9c. To investigate the absorption characteristic, we study the ZPL intensity which is defined as the area under the ZPL with a bandwidth of 10 meV (Fig. S9b). We observe a strong ZPL intensity at a detuning of 168 meV while an intermediate ZPL intensity is obtained at a detuning of 341 meV. These two important detunings are highlighted by horizontal lines in Fig. S9c and the corresponding photoluminescence spectra are shown in Fig. S9a. Another important detuning is exactly between the two aforementioned, i.e. at 255 meV, which yields a small ZPL intensity because it lays between the two states $|e,1\rangle$ and $|e,2\rangle$.

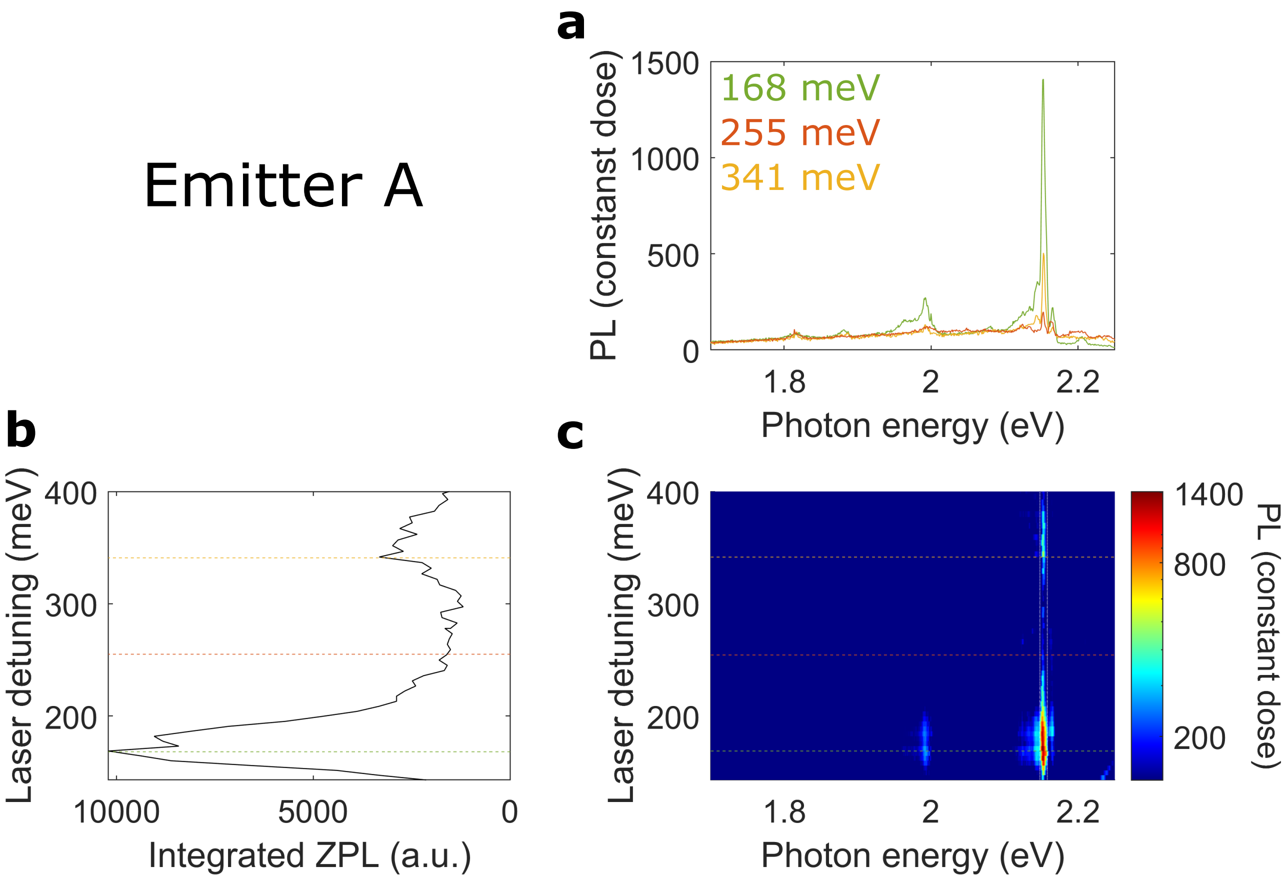

FIG. S9. **PLE of Emitter A at 10 K.** (a) Photoluminescence spectra of the luminescent centre at several laser detunings, given by the legend. (b) ZPL intensity as a function of laser detuning. The spectral range for the ZPL intensity is shown by the vertical lines in (c). (c) PLE map of a luminescent centre in hBN. The horizontal lines correspond to the spectra shown in (b) and the dim upward line in the bottom right corner corresponds to the silicon Raman $\sim 520\,\mathrm{cm}^{-1}$.

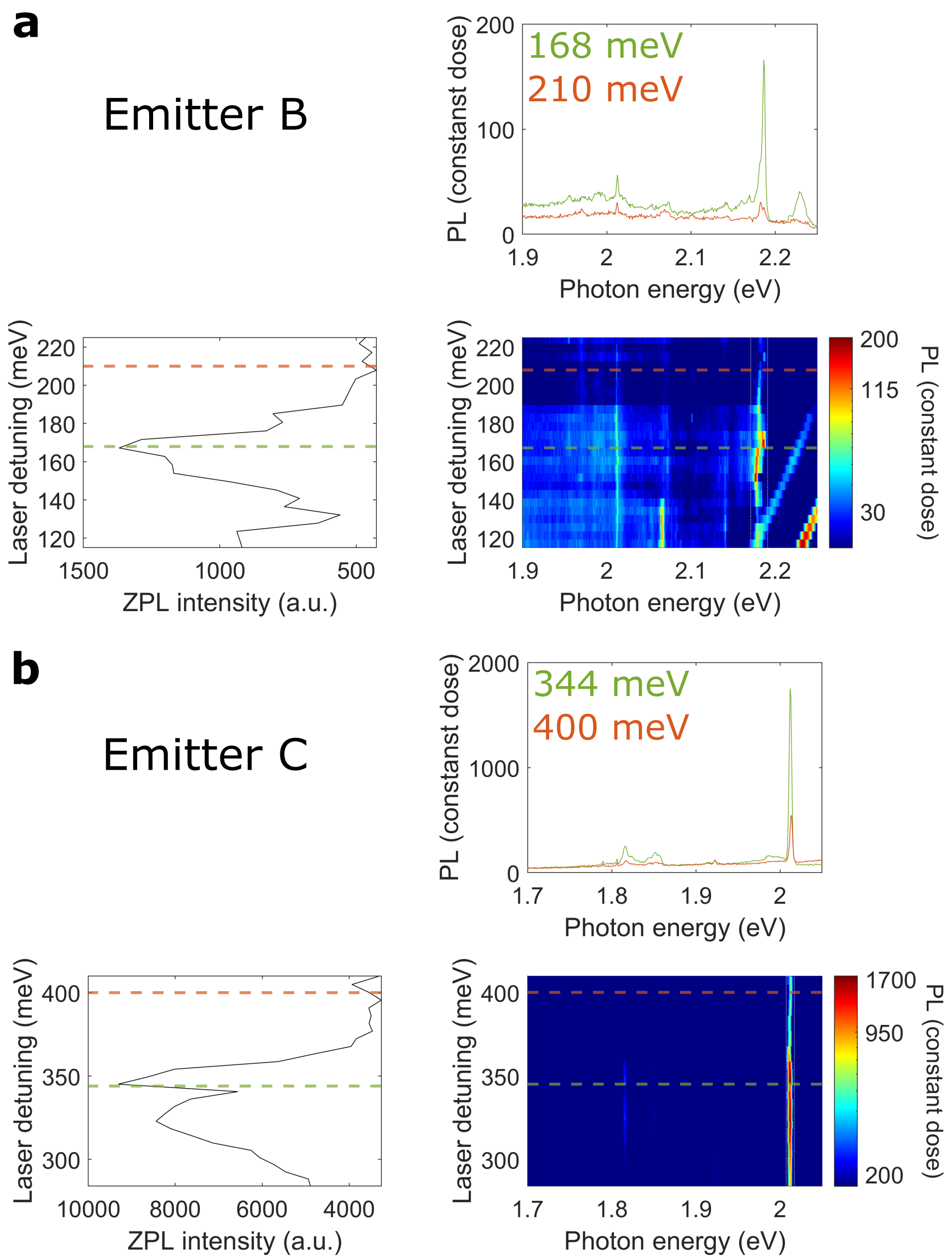

FIG. S10. **PLE of further luminescent centres at** 10 K**.** (a) PLE measurement of Emitter B, arranged and labelled as in Fig. S9. (b) PLE measurement of Emitter C, arranged and labelled as in Fig. S9.

VIII. RESCALING OF PLE SPECTRA FOR EMITTER A

The PLE measurements shown in Fig. 2a are done by two laser sweeps: Sweep 1 and Sweep 2. We use two different laser settings to keep the power between 0.75 and 1.93 mW (see Fig. S11a). We first describe the rescaling within one sweep, followed by the rescaling between two sweeps. We finally describe the rescaling to one excitation power.

We measure the excitation power as a function of excitation energy prior to the PLE measurements (Fig. S11a). Within one sweep, the exposure time at power P_i is set to

$$t_1 \cdot \frac{P_1}{P_i} \,, \tag{4}$$

where t_1 and P_1 are the exposure time and power at the beginning of the sweep while P_i is the current power (at a specific laser energy or laser detuning). This adjustment in exposure time is meaningful, since the luminescent centres studied show a linear dependence of the intensity with excitation power in the studied power range, as shown in Fig. S11d. Furthermore, the detector operates in a constant-count regime which avoids saturation.

At each laser detuning in Sweep 2, the exposure time is adjusted as described by equation (4). To rescale the spectra of Sweep 2 to Sweep 1, we multiply each spectrum of Sweep 2 (after adjusting the exposure time) by

$$\frac{t_1}{t_1^*} \cdot \frac{P_1}{P_1^*} \,, \tag{5}$$

where t_1^* and P_1^* are the exposure time and the excitation power at the beginning of Sweep 2.

We will give an example why this multiplication is meaningful. At the end of Sweep 2, the excitation energy is identical to the beginning of Sweep 1. However, the excitation power at the end of Sweep 2 is P^*_{end}. Now we calculate the exposure time at the end of Sweep 2. Calculating the exposure time of Sweep 2, we multiply equation (4) with equation (5) and get

$$t_1^* \cdot \frac{P_1^*}{P^*_{\text{end}}} \cdot \frac{t_1}{t_1^*} \cdot \frac{P_1}{P_1^*} = t_1 \cdot \frac{P_1}{P^*_{\text{end}}} \tag{6}$$

which is identical to the exposure time given by equation (4) where P_i is replaced by P^*_{end}.

After carrying out all the rescaling mentioned above, we rescale the data to 1 mW at an excitation energy of 2.431 eV. This is the reason why we have shown absolute values in the photoluminescence (i.e. constant dose) in Fig. 2.

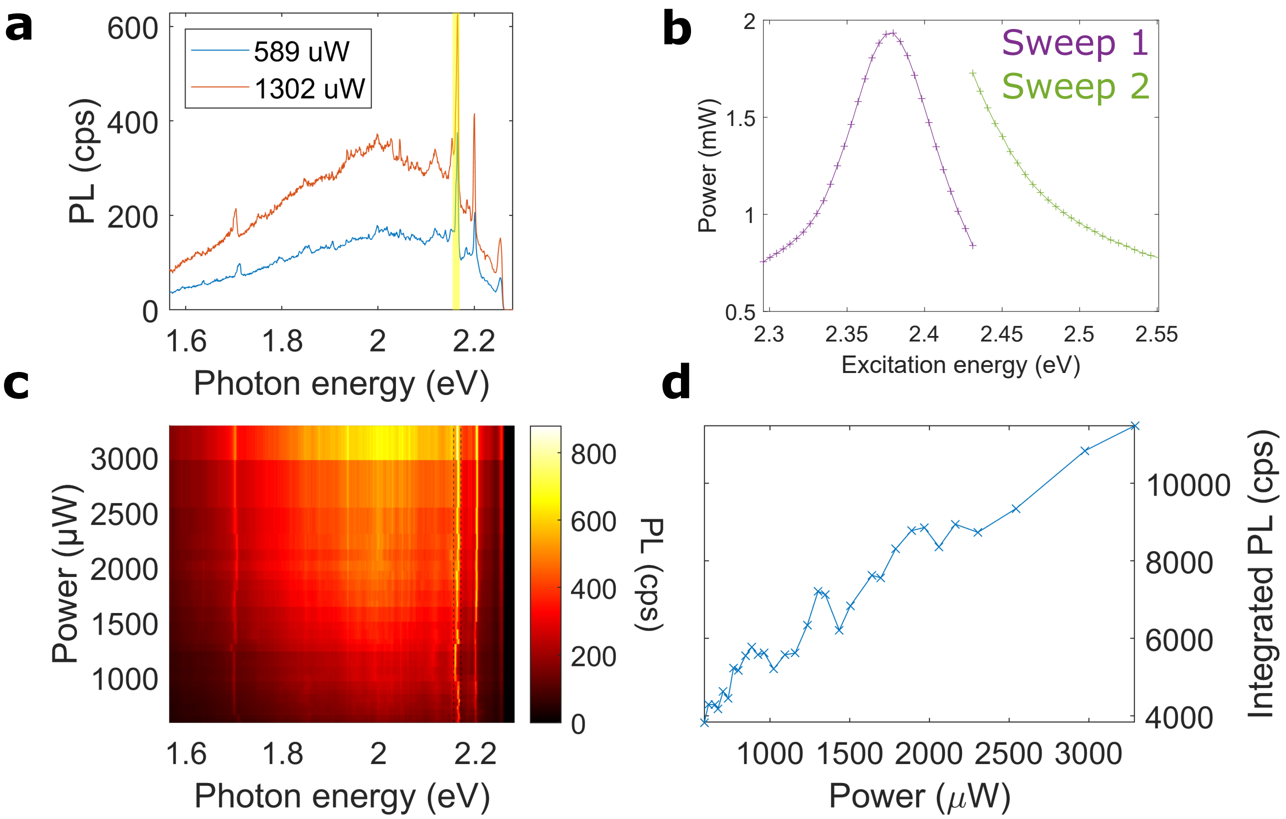

FIG. S11. **Power dependence of excitation source and photoluminescence.** (a) Photoluminesence spectra at several excitation powers, given by the legend. (b) The excitation power as a function of excitation energy. We performed two laser sweeps with different laser settings to obtain a similar power for all laser detunings. (c) Power-dependent photoluminescence. cps, counts per second. (d) Integrated zero-phonon line (ZPL) as a function of excitation power (not background corrected). The integrated spectral range (2.155 to 2.170 eV) is shown by vertical dashed lines in (c). We observe a linear dependence of the ZPL intensity as a function of excitation power.

IX. PLE OF OPTICAL AND ACOUSTIC PSB OF EMITTER A

Fig. S12a shows how background-corrected PLE intensities are obtained, by using the ZPL intensity as an example. We use an unrelated photoluminescence range with the same width as the ZPL to define a background intensity (grey curve in the central panel). To obtain the background-corrected ZPL intensity, we subtract this background intensity. The resulting background-corrected ZPL intensity is shown in the right panel of Fig. S12a. Similarly, we obtain the background-corrected intensities for several PSB, as shown in Fig. S12b and in the main text.

Fig. S12b shows the optical PSB intensities. The right panel shows that the optical PSB intensities at one-phonon detuning (170 meV) and the ZPL intensity at two-phonon detuning (340 meV) show comparable strength. All these processes are two-phonon processes (see Methods of the main text).

Fig. S12c shows the low-energy PSB at detunings around 10 meV. The low-energy PSB intensity is lower than for the ZPL, since it is a two-phonon process (see Methods and discussion in the main text). For the background-corrected intensities, we observe an almost identical qualitative behaviour of the low-energy PSB and ZPL intensity. This supports our interpretation that the low-energy PSB and the ZPL have the same excitation mechanism.

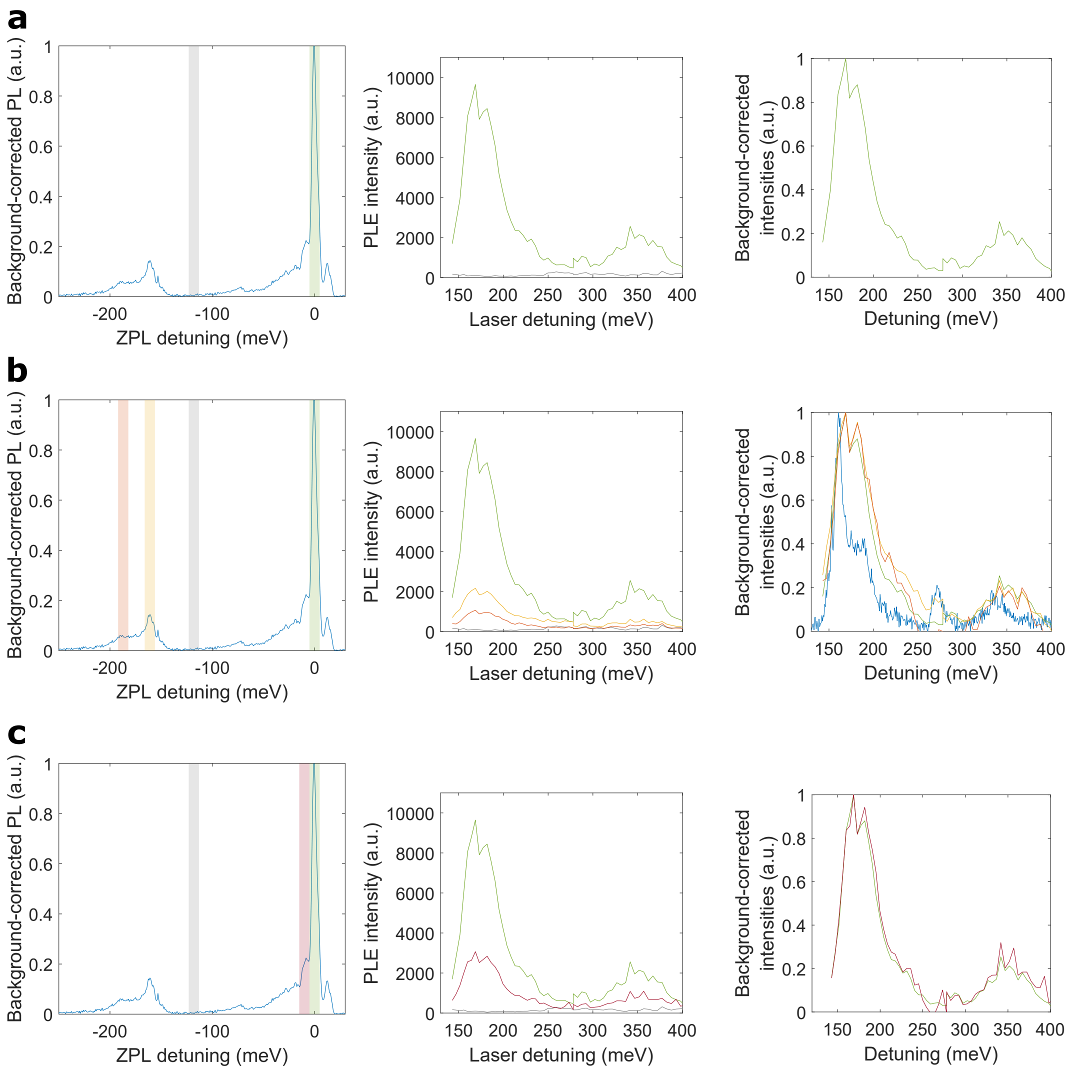

FIG. S12. **Background-correction of PLE intensities and acoustic PSB.** (a) Background-correction of the ZPL intensity. The spectral ranges of ZPL and background are shown in the left panel, on top of the photoluminescence spectrum. The resulting PLE intensities are shown in the central panel and the background-corrected ZPL intensity in the right panel. The latter is defined as the green ZPL intensity minus the grey background intensity. The photoluminescence spectrum in (a) is taken at a detuning of 168 meV. (b) Optical PSB intensities, labelled as in (a). In the right panel, the flipped photoluminescence spectrum is shown in blue. (c) Low-energy PSB intensity, labelling as in (a).

X. EXPERIMENTAL LINE SHAPES DIFFERENT TO GROUP I

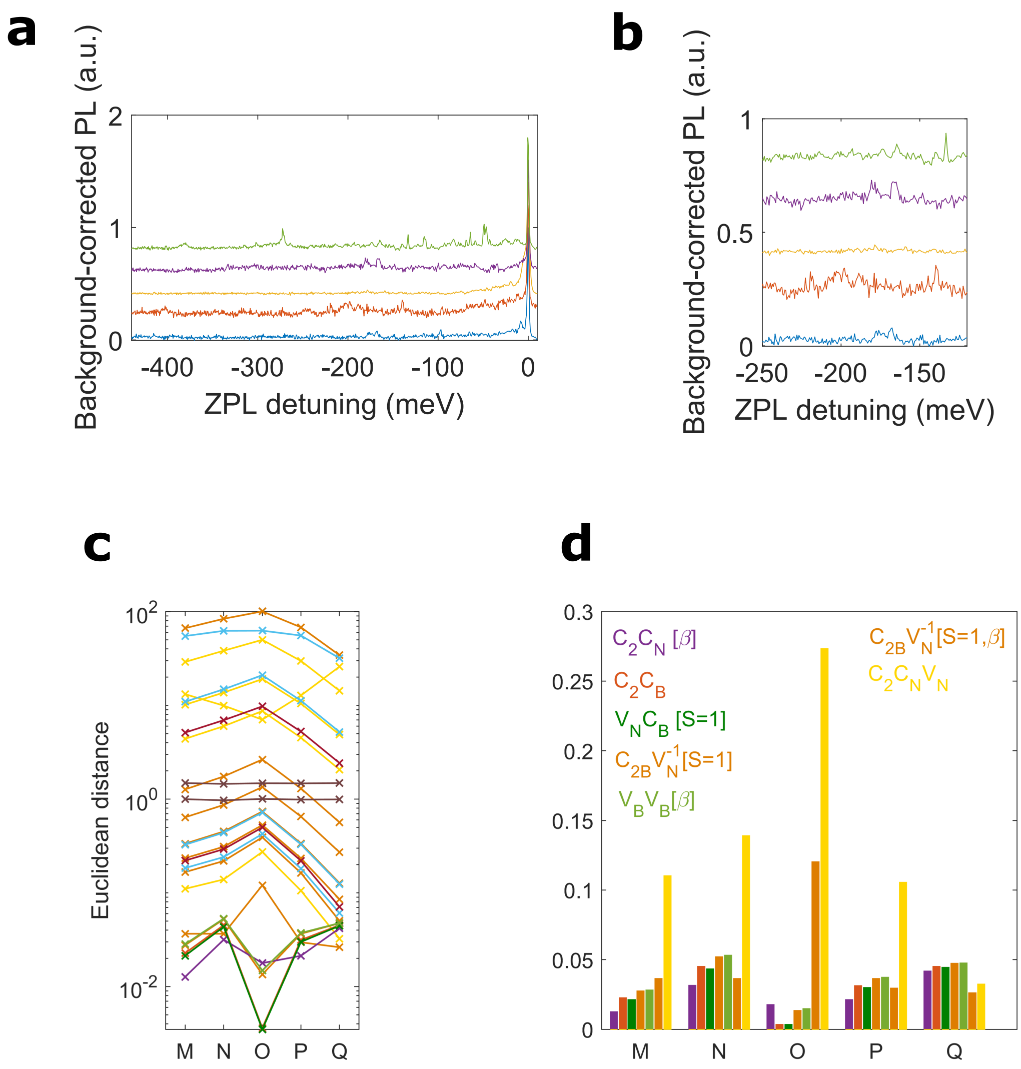

FIG. S13. **Low-temperature photoluminescence of luminescent centres not belonging to group I.** (a) Photoluminescence line shapes at $T = 10\,\mathrm{K}$ under 2.37 eV excitation, shifted vertically for clarity. The spectra from bottom to top correspond to Emitter M to Emitter Q, given by the x axis in (c). (b) Optical PSB of the luminescent centres shown in (a) with corresponding colors, shifted vertically for clarity. (c) Euclidean distance for Emitter M up to Emitter Q, with corresponding spectra shown in (a) from bottom to top. (d) Histograms of Emitter M to Emitter Q. The colors correspond to the defect transitions given by the legend. All line shapes are background corrected as outlined in Supplementary Information V and more details are given in the main text.

M	N	O	P	Q
2.0696	2.0139	2.1883	2.1210	2.1199

TABLE S3. Zero-phonon line energies (in electron-volt) of Emitter M to Q.

XI. OPTICAL CHARACTERISATION

For optical characterisation, we use an objective (details below) to focus a laser onto the sample. For photoluminescence (PL), we use a continuous-wave (CW) laser with a photon energy of 2.37 eV. A helium flow cryostat is used for the low temperature measurements.

For PLE measurements at 10 K, we use a supercontinuum white light laser (78 MHz repetition rate) which is filtered down to a bandwidth of ~ 1 nm with a tunable laser line filter. In order to compensate for intensity changes at different excitation wavelengths, we adjust the integration time of the spectrometer (see Supplementary Information VIII). For both photoluminescence (PL) and PLE measurements, the collected light is filtered by a longpass dichroic mirror (550 nm) and a 550 nm longpass filter. The filtered light is focused on the input slit of a spectrometer where the spectrum is obtained. The grating and detector information are given below.

For room temperature PL measurements, a 50X objective (NA = 0.6), a 150 lines/mm grating, and an EMCCD are used. The low-temperature PL and PLE experiments are performed using a home-made confocal microscope with a 50X objective (NA = 0.42), a 500 lines/mm grating, and a CCD detector. Furthermore, a spatial filter is used in front of the spectrometer for low-temperature measurements.

XII. MODELLING PHONON EFFECTS IN PHOTOLUMINESCENCE EMISSION FROM LUMINESCENCE CENTERS

A. The model Hamiltonian

We model the studied defect complex as a two level system (TLS) with ground state $|g\rangle$ and excited state $|e\rangle$, with splitting ω_e ($\hbar = 1$). The TLS is driven by a continuous wave laser with a frequency ω_L and Rabi coupling Ω. The emitter couples to both a vibrational and optical environment, characterised by the Hamiltonian [3]:

$$H(t) = \omega_e |e\rangle\langle e| + \Omega\cos(\omega_L t)\sigma_x + |e\rangle\langle e| \sum_{\mathbf{k}} g_k \left(b^\dagger_{\mathbf{k}} + b_{-\mathbf{k}}\right) + \sigma_x \sum_l \left(h^*_l a^\dagger_l + h_l a_l\right) + \sum_l \omega_l a^\dagger_l a_l + \sum_{\mathbf{k}} \nu_k b^\dagger_{\mathbf{k}} b_{\mathbf{k}}, \tag{7}$$

where we have defined $a^\dagger_l$ as the creation operator for a photon with energy ω_l, and $b^\dagger_{\mathbf{k}}$ as the creation operator for a phonon with energy $\nu_{\mathbf{k}}$ and wavevector $\mathbf{k}$. We have also introduced the system operators $\sigma_x = \sigma^\dagger + \sigma$ and $\sigma = |g\rangle\langle e|$. The coupling to the vibrational and electromagnetic environments are characterised by their respective spectral densities: for the optical environment we make the standard quantum optics approximation that the coupling constants h_l are frequency independent, such that $J_{\mathrm{EM}}(\omega) \approx \frac{\Gamma}{2\pi}$, where Γ is the emission rate of the optical transition; the phonon spectral density takes the form $J_{\mathrm{Ph}}(\nu) = \sum_{\mathbf{k}} S_{\mathbf{k}}\delta(\nu - \nu_{\mathbf{k}})$ where $S_{\mathbf{k}} = \omega^2_{\mathbf{k}}|g_{\mathbf{k}}|^2$ are the partial Huang-Rhys parameters, and contain contributions from phonons calculated through *ab initio* methods and those fitted to the emission as detailed below.

We can simplify the above equation by making the rotating-wave approximation and moving to a frame rotating with respect to the laser frequency ω_L, yielding the Hamiltonian:

$$H = \Delta |e\rangle\langle e| + \frac{\Omega}{2}\sigma_x + |e\rangle\langle e| \sum_k g_k (b^\dagger_k + b_k) + \sum_l \left(h^*_l \sigma a^\dagger_l e^{i\omega_L t} + h_l \sigma^\dagger a_l e^{-i\omega_L t}\right) + \sum_l \omega_l a^\dagger_l a_l + \sum_k \nu_k b^\dagger_k b_k, \tag{8}$$

where $\Delta = \omega_e - \omega_L$ is the detuning between the driving field and the exciton transition. This Hamiltonian forms the starting point of our analysis of the dynamical and optical properties of the defect.

B. Polaron theory for a driven emitter

In order to account for the strong coupling to the vibrational environment, we apply a polaron transformation to the global Hamiltonian, i.e. the unitary transformation $\mathcal{U}_P = |e\rangle\langle e| \otimes B_+ + |g\rangle\langle g|$, where $B_\pm = \exp\left(\pm \sum_{\mathbf{k}} \nu^{-1}_{\mathbf{k}} g_k (b^\dagger_{\mathbf{k}} - b_{-\mathbf{k}})\right)$ are displacement operators of the phonon environment [3, 5, 6]. This transformation dresses the excitonic states with vibrational modes of the phonon environment. In the polaron frame, the Hamiltonian may be written as $H_P = H_0 + H^{\mathrm{PH}}_{\mathrm{I}} + H^{\mathrm{EM}}_{\mathrm{I}}$, where

$$\begin{aligned} H_0 &= \tilde{\Delta} |e\rangle\langle e| + \frac{\Omega_R}{2}\sigma_x + \sum_l \omega_l a^\dagger_l a_l + \sum_k \nu_k b^\dagger_k b_k, \\ H^{\mathrm{PH}}_{\mathrm{I}} &= \frac{\Omega}{2}\left(\sigma_x B_x + \sigma_y B_y\right), \qquad \text{and} \qquad H^{\mathrm{EM}}_{\mathrm{I}} = \sum_l h^*_l \sigma B_+ a^\dagger_l e^{i\omega_L t} + \text{h.c.}, \end{aligned} \tag{9}$$

where we have introduced the phonon operators $B_x = (B_+ + B_- - 2B)/2$, $B_y = i(B_+ - B_-)/2$, and the Frank-Condon factor of the phonon environment $B = \mathrm{tr}_B(B_\pm \rho_B) = \exp(-(1/2)\sum_{\mathbf{k}} \nu^{-2}_{\mathbf{k}} |g_{\mathbf{k}}|^2 \coth(\nu_{\mathbf{k}}/k_B T))$, with the Gibbs state of the phonon environment in the polaron frame given by $\rho_B = \exp(-\sum_{\mathbf{k}} \nu_{\mathbf{k}} b^\dagger_{\mathbf{k}} b_{\mathbf{k}}/k_B T)/\mathrm{tr}\left(\exp(-\sum_{\mathbf{k}} \nu_{\mathbf{k}} b^\dagger_{\mathbf{k}} b_{\mathbf{k}}/k_B T)\right)$. Notice that the polaron transformation has dressed the operators in the light-matter coupling Hamiltonian, and renormalised the system parameters, such that $\Omega_R = \Omega B$ and $\tilde{\Delta} = \Delta - \sum_{\mathbf{k}} \nu^{-1}_{\mathbf{k}} g^2_{\mathbf{k}}$.

The dynamics of the electronic states of the emitter are described by a 2nd-order Born-Markov master equation [7], where both the electromagnetic field and the transformed vibrational environment are perturbatively eliminated. The polaron transformation means that the vibrational interaction is included non-perturbatively in the interaction strength [3]. We can use the fact that only terms quadratic in field operators are non-zero when traced with a Gibbs state, so that there will be no cross-terms between the vibrational and electromagnetic dissipators, such that the master equation can be written in two parts:

$$\frac{\partial \rho(t)}{\partial t} = -i\left[\tilde{\Delta} |e\rangle\langle e| + \frac{\Omega}{2}\sigma_x, \rho(t)\right] + \mathcal{K}_{\mathrm{PH}}[\rho(t)] + \frac{\Gamma}{2} L_\sigma[\rho(t)] + \frac{\gamma}{2} L_{\sigma^\dagger\sigma}, \tag{10}$$

where $\rho(t)$ is the reduced state of the emitter, $L_\sigma[\rho] = 2\sigma\rho\sigma^\dagger - \{\sigma^\dagger\sigma, \rho\}$ is the Lindblad form dissipator for the electromagnetic environment, and similarly $\mathcal{K}_{\rm PH}$ describes the dissipation due to the vibrational environment. Note that we have also included a source of homogeneous broadening given by rate γ, to account for experimental imperfections and other dephasing mechanisms such as charge noise. For the optical dissipator, we refer the reader to standard texts in quantum optics for the derivation of the optical component [7], in the following section we outline the derivation of the phonon dissipator.

1. *Deriving the phonon dissipator*

Considering only the coupling to the vibrational modes, we move into the interaction picture with respect to the Hamiltonian H_0, the interaction term becomes:

$$H_{\rm I}^{\rm PH}(t) = \frac{\Omega}{2}[\sigma_x(t)B_x(t) + \sigma_y(t)B_y(t)], \tag{11}$$

where the interaction picture system operators are given by:

$$\begin{aligned}\sigma_x(t) &= \eta^{-2}\left[\tilde{\Delta}\Omega_R(1-\cos(\eta t))\sigma_z + (\Omega_R^2 + \tilde{\Delta}^2\cos(\eta t))\sigma_x + \tilde{\Delta}\eta\sin(\eta t)\sigma_y\right],\\ \sigma_y(t) &= \eta^{-1}\left(\Omega_R\sin(\eta t)\sigma_z + \eta\cos(\eta t)\sigma_y - \tilde{\Delta}\sin(\eta t)\sigma_x\right),\end{aligned} \tag{12}$$

and we have defined the generalised Rabi frequency $\eta = \sqrt{\tilde{\Delta}^2 + \Omega_R^2}$. The interaction picture bath operators are found as $B_{x/y}(t) = e^{iH_0t}B_{x/y}e^{-iH_0t}$, with $B_{x/y}$ defined above. In the Schrödinger picture and making the Born-Markov approximation in the polaron frame [7], the dissipator describing the electron-phonon interaction is given by:

$$\mathcal{K}_{\rm PH}[\rho(t)] = -\frac{\Omega^2}{4}\int_0^\infty \left([\sigma_x, \sigma_x(-\tau)\rho(t)]\Lambda_{xx}(\tau) + [\sigma_y, \sigma_y(-\tau)\rho(t)]\Lambda_{yy}(\tau) + \text{h. c.}\right)d\tau, \tag{13}$$

where we have and introduced the phonon bath correlation functions as $\Lambda_{xx}(\tau) = \langle B_x(\tau)B_x\rangle = B^2(e^{\varphi(\tau)} + e^{-\varphi(\tau)} - 2)$, $\Lambda_{yy}(\tau) = \langle B_y(\tau)B_y\rangle = B^2(e^{\varphi(\tau)} - e^{-\varphi(\tau)})$ which quantify the response of the phonon environment to the exciton dynamics. Taking the continuum limit over the phonon modes, these correlation functions can be described in terms of the polaron frame propagator $\varphi(\tau) = \int_0^\infty d\nu\nu^{-2}J(\nu)[\coth(\nu/2k_{\rm B}T)\cos(\nu\tau) - i\sin(\nu\tau)]$. We can simplify the form of the master equation by evaluating the integrals over τ, leading to:

$$\mathcal{K}_{\rm PH}[\rho_s(t)] = -\frac{\Omega^2}{4}\left([\sigma_x, \chi_x\rho_s(t)] + [\sigma_y, \chi_y\rho_s(t)] + \text{h. c.}\right), \tag{14}$$

where we have introduced the rate operators:

$$\chi_x = \int_0^\infty \sigma_x(-\tau)\Lambda_{xx}(\tau)d\tau = \frac{1}{\eta^2}\left[\Delta\Omega_r(\Gamma_0^x - \Gamma_c^x)\sigma_z + (\Omega_r^2\Gamma_0^x + \Delta^2\Gamma_c^x)\sigma_x + \Delta\eta\Gamma_s^x\sigma_y\right], \tag{15}$$

$$\chi_y = \int_0^\infty \sigma_y(-\tau)\Lambda_{yy}(\tau)d\tau = \frac{1}{\eta}\left[\Omega_r\Gamma_s^y\sigma_z - \Delta\Gamma_s^y\sigma_x + \eta\Gamma_c^y\sigma_y\right], \tag{16}$$

and defined the terms $\Gamma_0^a = \int_0^\infty \Lambda_{aa}(\tau)d\tau$, $\Gamma_c^a = \int_0^\infty \Lambda_{aa}(\tau)\cos(\eta\tau)d\tau$, $\Gamma_s^a = \int_0^\infty \Lambda_{aa}(\tau)\sin(\eta\tau)d\tau$, with $a \in \{x, y\}$. We may understand these terms as the rates at which transitions occur between the eigenstates of the system (i.e. the dressed states) induced by phonons. Qualitatively, Γ_s^a and Γ_c^a describe processes where a polaron is formed during excitation of the emitter, resulting in an exchange of energy with the environment. In contrast, Γ_0^a leads to no net exchange of energy, inducing a pure dephasing type process.

Since we are operating in the polaron frame, the above master equation is non-perturbative in the electron-phonon coupling strength, capturing strong-coupling and non-Markovian influences in the lab frame, despite having made a Born Markov approximation. This provides us with a simple, intuitive, and computationally straight-forward method for describing exciton dynamics in a regime where a standard weak coupling master equation would break down [3].

C. Correlation functions and spectra for a driven emitter

We are interested in understanding the impact that phonon coupling has on the PLE spectrum, which can be calculated from the first-order correlation function $g^{(1)}(t,\tau)$. As shown in Ref. [8], the first-order correlation function in the polaron frame for the emitter is written in terms of the polaronic dipole operator $\underline{\boldsymbol{\sigma}} = \sigma B_+$, that is, the correlation function is $g_0^{(1)}(t,\tau) = \langle B_-(t+\tau)\sigma^\dagger(t+\tau)B_+(t)\sigma(t)\rangle$. In general, $B_\pm$ are many-body displacement operators that do not commute with system operators. However, in the regime that the polaron master equation is valid at second-order, we can factor this correlation function in the Born approximation, such that $g_0^{(1)}(t,\tau) \approx \mathcal{G}(\tau)g_{\rm opt}^{(1)}(t,\tau)$. There are two terms in this expression, the correlation function for the scattered field $g_{\rm opt}^{(1)}(t,\tau) = \langle\sigma^\dagger(t+\tau)\sigma(t)\rangle$ describing the purely electronic transitions, and the phonon correlation function $\mathcal{G}(\tau) = \langle B_-(\tau)B_+\rangle = B^2\exp(\varphi(\tau))$ which accounts for the relaxation of the phonon environment. This factorisation allows us to split the emission spectrum into two components $S(\omega) = S_{\rm opt}(\omega) + S_{\rm SB}(\omega)$, where $S_{\rm opt}(\omega) = {\rm Re}[B^2\int_0^\infty dt\int_0^\infty g_{\rm opt}^{(1)}(t,\tau)e^{i\omega\tau}d\tau]$ corresponds to purely optical transitions, and $S_{\rm SB}(\omega) = {\rm Re}[\int_0^\infty dt\int_0^\infty(\mathcal{G}(\tau)-B^2)g_{\rm opt}^{(1)}(t,\tau)e^{i\omega\tau}d\tau]$ is attributed to non-Markovian phonon relaxation during the emission process, and leads to the emergence of a phonon sideband [9].

[1] A. Sajid, J. R. Reimers, and M. J. Ford, Defect states in hexagonal boron nitride: Assignments of observed properties and prediction of properties relevant to quantum computation, Phys. Rev. B **97**, 064101 (2018).
[2] M. K. Svendsen, S. Ali, N. Stenger, K. S. Thygesen, and J. Iles-Smith, Signatures of non-Markovianity in cavity-QED with color centers in 2D materials (2022), arXiv:2207.10630.
[3] A. Nazir and D. P. S. McCutcheon, Modelling exciton–phonon interactions in optically driven quantum dots, Journal of Physics: Condensed Matter **28**, 103002 (2016).
[4] Background correction, `https://in.mathworks.com/matlabcentral/fileexchange/27429-background-correction?s_tid=FX_rc2_behav` (2012), accessed: 30.03.2022.
[5] D. P. McCutcheon and A. Nazir, Quantum dot Rabi rotations beyond the weak exciton–phonon coupling regime, New Journal of Physics **12**, 113042 (2010).
[6] J. Iles-Smith, D. P. McCutcheon, J. Mørk, and A. Nazir, Limits to coherent scattering and photon coalescence from solid-state quantum emitters, Physical Review B **95**, 201305 (2017).
[7] H. Breuer, F. Petruccione, and S. Petruccione, *The Theory of Open Quantum Systems* (Oxford University Press, 2002).
[8] J. Iles-Smith, D. P. McCutcheon, A. Nazir, and J. Mørk, Phonon scattering inhibits simultaneous near-unity efficiency and indistinguishability in semiconductor single-photon sources, Nature Photonics **11**, 521 (2017).
[9] A. J. Brash, J. Iles-Smith, C. L. Phillips, D. P. McCutcheon, J. O'Hara, E. Clarke, B. Royall, L. R. Wilson, J. Mørk, M. S. Skolnick, *et al.*, Light scattering from solid-state quantum emitters: beyond the atomic picture, Physical Review Letters **123**, 167403 (2019).
